# CCDC80 Protects against Aortic Dissection and Rupture by Maintaining the Contractile Smooth Muscle Cell Phenotype

**DOI:** 10.1002/advs.202502108

**Published:** 2025-04-25

**Authors:** Qingqing Xiao, Yi Li, Bin Cai, Xiying Huang, Liang Fang, Feng Liang, Long Chen, Ke Xu, Weifeng Zhang, Xiaolei Wang, Anwen Yin, Xia Wang, Zhaohua Cai, Fei Zhuang, Qin Shao, Bin Zhou, Berthold Hocher, Ben He, Linghong Shen

**Affiliations:** ^1^ Department of Cardiology Shanghai Chest Hospital Shanghai Jiao Tong University School of Medicine Shanghai 200030 China; ^2^ Department of Cardiology Shanghai General Hospital Shanghai Jiao Tong University School of Medicine Shanghai 200080 China; ^3^ Department of Rheumatology Peking Union Medical College Hospital Peking Union Medical College & Chinese Academy of Medical Sciences Beijing 100730 China; ^4^ Department of Cardiac Surgery Shanghai Chest Hospital Shanghai Jiaotong University School of Medicine Shanghai 200030 China; ^5^ Chinese Academy of Sciences University of Chinese Academy of Sciences Shanghai 200032 China; ^6^ Fifth Department of Medicine (Nephrology/Endocrinology/Rheumatology/Pneumology) University Medical Centre Mannheim University of Heidelberg 69123 Heidelberg Germany; ^7^ Reproductive and Genetic Hospital of CITIC‐Xiangya, People's Republic of China Changsha 410028 China; ^8^ IMD Institut fur Medizinische Diagnostik Berlin‐Potsdam GbR 14473 Berlin Germany

**Keywords:** aortic dissection, CCDC80, JAK2/STAT3 signaling pathway, vascular remodeling, VSMC phenotype switching

## Abstract

Aortic dissection (AD) is a life‐threatening medical emergency characterized by adverse vascular remodeling. Coiled‐coil domain‐containing protein 80 (CCDC80) plays an essential role in regulating cardiovascular remodeling. This study aims to define the role of CCDC80 in the formation and development of AD. Significant downregulation of CCDC80 in vascular smooth muscle cell (VSMC) in human and mouse AD is identified. Then, CCDC80 knockout mice (CCDC80^−/−^) and VSMC‐specific CCDC80 knockout mice (CCDC80^fl/fl^ SM22α Cre^+^) treated with angiotensin II (Ang II) or Ang II combined with β‐aminopropionitrile monofumarate (BAPN) frequently develop AD with higher frequency and severity, accompanied by severe elastin fragmentation and collagen deposition. Mechanistically, CCDC80 interacts with JAK2, and CCDC80 deficiency promotes VSMC phenotype switching, proliferation, and migration as well as matrix metalloproteinase production by activating the JAK2/STAT3 signaling pathway. Moreover, the JAK2/STAT3 pathway‐specific inhibitor ameliorates adverse vascular remodeling and reduces AD formation in CCDC80‐knockout mice by mitigating VSMC phenotype switching. In conclusion, CCDC80 deficiency exacerbates the progression of events leading to AD by activating the JAK2/STAT3 pathway involved in regulating the phenotype switching and function of VSMCs. These findings highlight that CCDC80 is a potential key target for the prevention and treatment of AD.

## Graphical abstract

Aortic dissection (AD) is accompanied by a decrease in CCDC80 in vascular smooth muscle cells (VSMCs). CCDC80 can interact with JAK2, and VSMC‐specific CCDC80 deficiency accelerates the progression of AD by activating the JAK2/STAT3 pathway involved in regulating the phenotype switching and function of VSMCs. These findings suggest that CCDC80 is a promising therapeutic target for AD.

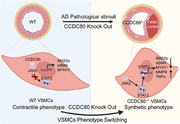

## 1 Introduction

Aortic dissection (AD) is a life‐threatening medical emergency with a high mortality rate and is characterized by a tear in the aortic intima or bleeding within the aortic wall.^[^ ^1^ ^]^ Major risk factors for AD include hypertension, dyslipidemia, and autoimmune vascular diseases.^[^ ^2^ ^]^ Clinically, AD management mainly includes open surgery, endovascular interventions (e.g., thoracic endovascular aortic repair) and pharmacological therapies (e.g., β‐blockers, other antihypertensives).^[^ ^3^, ^4^ ^]^ The pathological mechanism of AD is currently unclear, and effective treatment strategies need to be explored to prevent or delay the progression of AD.

Vascular smooth muscle cell (VSMC) phenotype switching plays a key role in the pathogenesis of several cardiovascular diseases, such as atherosclerosis, post‐injury restenosis, aneurysm, and AD.^[^ ^5^, ^6^, ^7^ ^]^ VSMC phenotype switching from a contractile to synthetic phenotype is an early event in the development of aortic aneurysms and AD.^[^ ^8^, ^9^, ^10^ ^]^ This includes the downregulation of contractile phenotype markers in VSMCs: smooth muscle 22 α (SM22α) and α‐smooth muscle actin (α‐SMA), promoting VSMC proliferation, migration, and inflammation and matrix metalloproteinase (MMP) secretion, thereby leading to the degradation of the extracellular matrix (ECM); this subsequently weakens the aortic wall and contributes to aortic rupture.^[^ ^11^, ^12^ ^]^ However, the specific mechanism of VSMC phenotype switching in AD remains unelucidated.

Our previous study has suggested that exercise‐derived exosomal CCDC80tide ameliorates angiotensin II (Ang II)‐induced pathological myocardial remodeling.^[^ ^13^ ^]^ The preprotein of CCDC80tide—coiled‐coil domain‐containing protein 80 (CCDC80, also known as URB, SSG1, and DRO1), a member of the coiled‐coil domain‐containing protein family—is mainly expressed in VSMCs.^[^ ^14^ ^]^ CCDC80 plays an integral role in the regulation of cardiovascular remodeling and homeostasis.^[^ ^14^, ^15^, ^16^ ^]^ Recent study demonstrated that CCDC80 could accelerate atherosclerosis by decreasing lipoprotein lipase expression.^[^ ^15^ ^]^ Clinical study has found that CCDC80 is a risk gene locus for familial intracranial aneurysms in the French‐Canadian population.^[^ ^17^ ^]^ However, the specific role of CCDC80 in VSMC phenotype switching and the progression of AD remains unknown.

In the present study, we aimed to investigate whether CCDC80 was involved in the pathogenesis of AD and examine the underlying mechanisms. We found that CCDC80 deficiency exacerbated the progression of AD by activating the JAK2/STAT3 pathway involved in regulating the phenotype switching and function of VSMCs. Therefore, our data indicated that CCDC80 is a potential target for the prevention and treatment of AD.

## 2 Results

### 2.1 CCDC80 Expression Was Significantly Reduced in Human and Mouse AD

**Figure 1:**
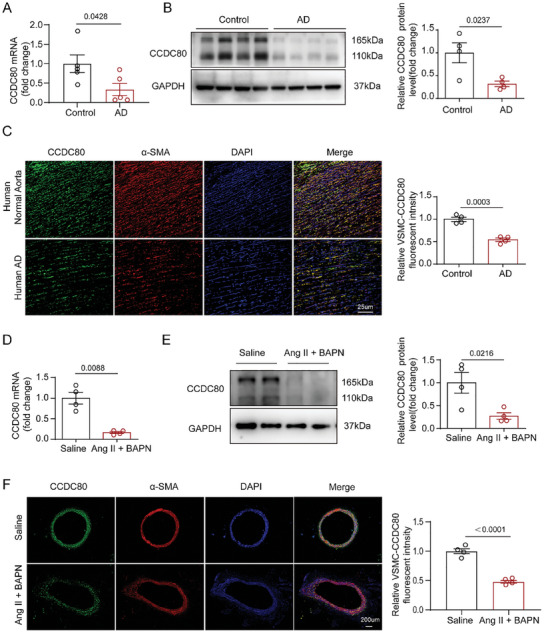
CCDC80 expression is downregulated in human and mouse AD. A) Relative mRNA levels of CCDC80 in aortic tissues from non‐AD controls and patients with AD, *n* = 5 per group. B) Western blotting and quantification of CCDC80 levels in the aortas of non‐AD controls and patients with AD, *n* = 4 per group. C) Representative images of immunofluorescence staining for CCDC80 (green), α‐SMA (red), and DAPI (blue) in the ascending thoracic aortic media of controls and patients with AD; scale bar = 25 µm. Quantification of CCDC80‐positive area in VSMCs in the right panel, *n* = 4 per group. D) Relative mRNA levels of CCDC80 in aortic tissues from healthy controls and AD mice, *n* = 4 per group. E) Western blotting and quantification of CCDC80 levels in healthy controls and AD mice, *n* = 4 per group. F) Representative images of immunofluorescence staining of CCDC80 (green), α‐SMA (red), and DAPI (blue) in aortic tissues from mice with AD and sham controls; scale bar = 200 µm. Quantification of CCDC80‐positive area in VSMCs in the right panel, *n* = 4 per group. Data are presented as mean ± SEM. Statistical analysis was performed using Student's t‐test (A,B,E,F). Student's t‐test with Welch's correction (D).

First, we examined the expression of CCDC80 in different tissues of adult mice. CCDC80 protein was expressed in several tissues including the lung, brain, and aorta; it was particularly expressed in the aorta (FigureS1A, Supporting Information), which is consistent with a recent report.^[^ ^14^ ^]^ Remarkably, a high expression of CCDC80 protein was observed in VSMCs and endothelial cells but not in adventitial fibroblasts and macrophages in the aorta (Figure S1B, Supporting Information). Moreover, its expression decreased with age in male mice (Figure S1C, Supporting Information). These data indicate that CCDC80 is highly enriched in aortic VSMCs and may play an important role in maintaining vascular homeostasis.

To examine the role of CCDC80 in the aorta, we examined CCDC80 expression in the ascending aorta (ASC) of patients and mice with AD. CCDC80 mRNA and protein levels were downregulated in human AD aortas (**Figure** **1**A,B). Immunofluorescence (IF) staining showed that in human AD, CCDC80 was mainly reduced in VSMCs but not in endothelial cells (Figure 1C and Figure S2, Supporting Information). Moreover, quantitative PCR (qPCR) analysis (Figure 1D) and western blotting (WB; Figure 1E) showed that CCDC80 expression decreased in the aorta of Ang II + β‐aminopropionitrile monofumarate (BAPN)‐induced AD mice. Similarly, IF staining showed that CCDC80 was reduced in VSMCs of AD mice (Figure 1F). Collectively, these data demonstrate that CCDC80 was downregulated in both human and mice AD VSMCs, thereby indicating that CCDC80 plays a role in AD.

### 2.2 Global CCDC80 Knockout Mice Exacerbated AD Formation and Rupture

To investigate the role of CCDC80 in AD, we performed in vivo experiments using a mouse model of Ang II + BAPN‐induced AD. First, global CCDC80 knockout mice (CCDC80^−/−^) were generated by inducing a CCDC80 genetic mutation. The global CCDC80 knockout mutation was confirmed at DNA, mRNA, and protein levels (Figure S3, Supporting Information). Then, CCDC80^−/−^ mice and C57BL/6J background mice (wild‐type mice, WT) were treated with Ang II + BAPN for 28 d. During the 28 d Ang II + BAPN treatment, CCDC80^−/−^ mice developed AD with increased frequency and severity compared with their littermate controls (55.56% (4/9) versus 100.00% (11/11), *p* = 0.0260) (Figure S4A,C, Supporting Information). The incidence of WT mice dying of AD and rupture was 11.11% (1/9). By contrast, 72.73% (8/11) of CCDC80^−/−^ mice died of aortic rupture (Figure S4B,C, Supporting Information). Blood pressure levels were similar between Ang II + BAPN‐treated CCDC80^−/−^ and WT mice (Table S1, Supporting Information). Vascular ultrasound imaging and maximal aortic diameter measurement at 28 days after vascular modeling demonstrated that compared with WT controls, CCDC80^−/−^ mice exacerbated Ang II + BAPN‐induced aortic dilation (Figure S4D–F, Supporting Information). These findings demonstrate that CCDC80 deficiency significantly increased aortic dilatation and promoted AD formation and rupture in mice.

**Figure 2:**
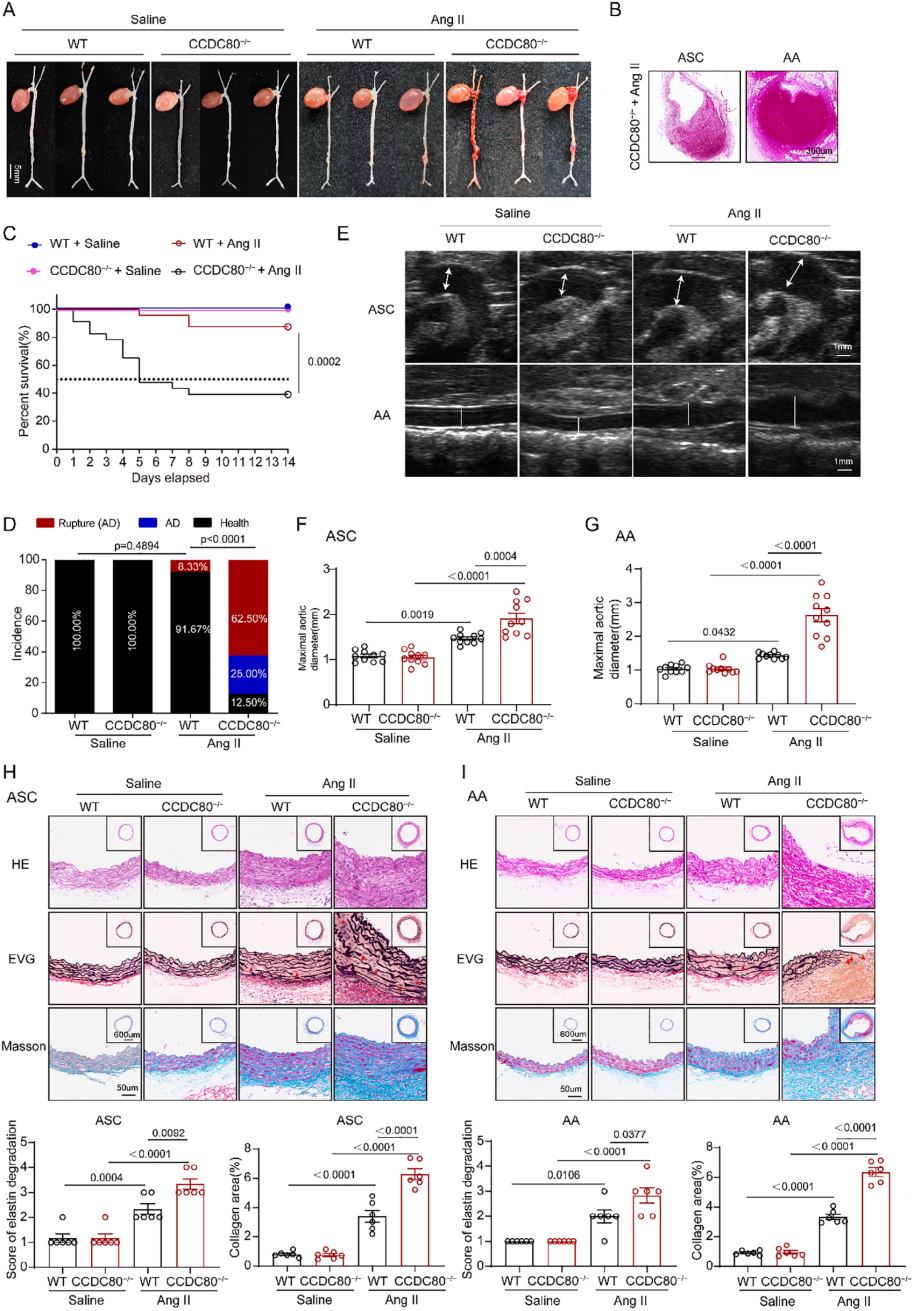
CCDC80 deficiency predisposes to aortic dissection and rupture in Ang II‐treated mice. A–H) WT and CCDC80^−/−^ mice were treated with saline or Ang II for 14 d. A) Representative wholemount images of aortas showing rupture and hematoma. B) Representative transverse section of the dissected ascending aorta (ASC) and abdominal aorta (AA) from Ang II‐treated CCDC80^−/−^ mice stained with hematoxylin and eosin (H&E). C) Kaplan–Meier survival curves of male WT and CCDC80^−/−^ mice during 14 d of saline or Ang II administration, *n* = 24 per group. D) Quantification of incidence (AD, rupture, and health) in entire aortas from WT and CCDC80^−/−^ mice, *n* = 24 per group. E) Representative ultrasound images of ASC and AA. F,G) Measurements of maximum ASC and AA, WT mice and CCDC80^−/−^ mice, *n* = 10 per group. H,I) Representative transverse sections of H) ASC and I) AA from saline or Ang II‐treated and CCDC80^−/−^ mice stained with H&E, Masson's trichrome blue, and EVG staining. Red arrows indicate the rupture of elastin fibers. The grades of elastin degradation and collagen deposition in the aortic wall of mice were measured, *n* = 6 per group. Data are presented as mean ± SEM. Statistical analysis was performed using the Kaplan–Meier method and compared using log‐rank tests for C, A Fisher's exact test for D, and 2‐way ANOVA with Tukey's post hoc test for F–I.

Hypertension is the most important risk factor for AD.^[^ ^18^ ^]^ Approximately 80% of patients with AD experience hypertension.^[^ ^19^, ^20^ ^]^ Patients susceptible to the occurrence of AD primarily manifest elevated maximum systolic and mean aortic blood pressure levels.^[^ ^21^ ^]^ To evaluate the effect of hypertension on CCDC80^−/−^ mice, we injected Ang II (1000 ng/kg/min) into male CCDC80^−/−^ and WT control mice for 14 d. Blood pressure was elevated in Ang II‐injected mice and was similar in Ang II‐injected CCDC80^−/−^ and WT mice (Table S2, Supporting Information). Remarkably, during the 14 d Ang II administration, 87.50% (21/24) of CCDC80^−/−^ mice experienced AD following Ang II treatment—primarily in the ASC and suprarenal abdominal aorta (AA)—compared with 8.33% (2/24) of WT mice (*p* < 0.0001) (**Figure** **2**A,D); 62.50% (15/24) of male CCDC80^−/−^ mice died of AD and rupture (Figure 2C,D) compared with 8.33% (2/24) of male WT mice. Moreover, WT and CCDC80^−/−^ mice treated with saline did not develop AD (Figure 2A,D). Dissected aortas in CCDC80^−/−^ mice exhibited a compressed true lumen as well as either an intramural hematoma or a large false lumen with thrombosed blood (Figure 2B). Vascular ultrasound imaging and maximal aortic diameter measurement performed on day 14 after modeling demonstrated that CCDC80 knockout exacerbated Ang II‐induced aortic dilation (Figure 2E–G). Hematoxylin and eosin (H&E), elastic van Gieson (EVG) staining, and Masson's staining demonstrated that AD formation, collagen deposition, elastic disarray, and elastic fiber degradation were exacerbated in Ang II‐treated CCDC80^−/−^ mice compared with WT mice (Figure 2H,I). Therefore, CCDC80 deficiency increases susceptibility to AD and rupture.

### 2.3 VSMC‐Specific CCDC80 Ablation Exacerbated AD Formation and Rupture in Mice

VSMCs are the main component cells of the vascular wall. They play an important role in maintaining vascular tone and integrity, regulating intravascular pressure, and redistributing blood volume, which are crucial for maintaining vascular homeostasis.^[^ ^22^, ^23^ ^]^ CCDC80 was dramatically reduced in the aortas of patients and mice with AD primarily in VSMCs (Figure S1, Supporting Information). Therefore, we constructed VSMC‐specific CCDC80 knockout mice to examine the role of CCDC80 in AD.

To determine the role of VSMC‐specific CCDC80 in AD, we generated conditional CCDC80 knockout mice in VSMC (CCDC80^fl/fl^ SM22α Cre^+^) by crossing mice in which the exon 3 of the CCDC80 gene was flanked by two LoxP sites (CCDC80^fl/fl^) with the mice containing the SM22α Cre^+^ mutation (Figure S5A, Supporting Information). The VSMC‐specific deletion of CCDC80 was confirmed at DNA, mRNA, and protein levels (Figure S5B–F, Supporting Information). There were no significant differences in expression in other tissues (heart, liver, lung, kidney, muscle, and brain) between CCDC80^fl/fl^ SM22α Cre^−^ and CCDC80^fl/fl^ SM22α Cre^+^ mice (Figure S5C, Supporting Information).

**Figure 3:**
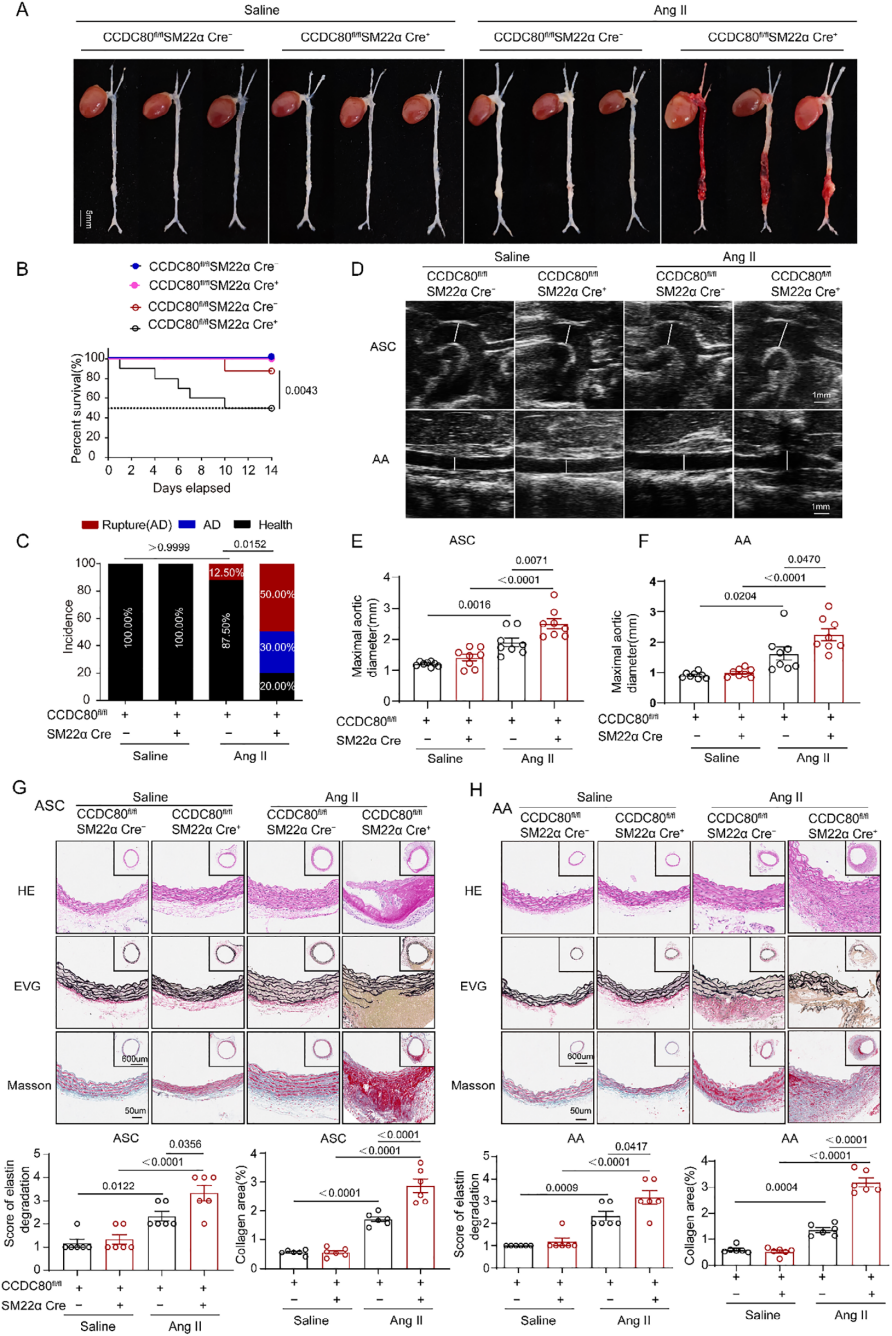
Mice with an VSMC‐specific CCDC80 deletion are susceptible to Ang II‐induced AD. A–H) CCDC80^fl/fl^SM22α Cre^−^ and CCDC80^fl/fl^SM22α Cre^+^ mice were treated with saline or Ang II for 14 d. A) Representative wholemount images of saline or Ang II‐treated CCDC80^fl/fl^SM22α Cre^−^ and CCDC80^fl/fl^SM22α Cre^+^ aortas showing rupture and hematoma in the aortas. B) Kaplan–Meier survival curves of male CCDC80^fl/fl^SM22α Cre^−^ and CCDC80^fl/fl^SM22α Cre^+^ mice during 14 d of saline or Ang II administration (*n* = 8–10 per group). C) Quantification of incidence (AD, rupture, and health) in whole aortas from CCDC80^fl/fl^SM22α Cre^−^ and CCDC80^fl/fl^SM22α Cre^+^ mice. D) Representative ultrasound images of the ascending aorta (ASC) and abdominal aorta (AA); scale bar = 1 mm. E,F) Measurements of maximum ASC and AA, WT mice and CCDC80^−/−^ mice (*n* = 8 per group). G,H) Representative transverse sections of G) ASC and H) AA from saline or Ang II‐treated CCDC80^fl/fl^SM22α Cre^−^ and CCDC80^fl/fl^SM22α Cre^+^ mice stained with H&E, Masson's trichrome blue, and EVG. The grades of elastin degradation and collagen deposition in the aortic wall of mice were measured (*n* = 6 per group). Data are presented as mean ± SEM. Statistical analysis was performed using the Kaplan–Meier method and compared using the log‐rank test for B, A Fisher's exact test for C, and 2‐way ANOVA with Tukey's post hoc test for E–H.

Next, we administered saline or Ang II to CCDC80^fl/fl^ SM22α Cre^+^ and CCDC80^fl/fl^ SM22α Cre^−^ mice for 14 days. Blood pressure levels were similar between Ang II‐injected CCDC80^fl/fl^ SM22α Cre^+^ and CCDC80^fl/fl^ SM22α Cre^−^ mice (Table S3, Supporting Information). Although Ang II treatment resulted in AD in both genotypes, the incidence was significantly higher in CCDC80^fl/fl^ SM22α Cre^+^ mice than in CCDC80^fl/fl^ SM22α Cre^−^ mice (80.00% versus 12.50%, *p* < 0.01) (**Figure** **3**A,C). AD particularly developed in the ASCs and AAs in VSMC‐specific CCDC80 knockout mice (Figure 3A). Furthermore, a higher frequency of aortic rupture was noted in CCDC80^fl/fl^ SM22α Cre^+^ mice (5/10, 50.00%) compared with that in CCDC80^fl/fl^ SM22α Cre^−^ mice (1/8, 12.50%; Figure 3B,C). After 2 weeks of Ang II treatment, vascular ultrasound imaging and maximal aortic diameter measurement demonstrated that VSMC‐specific CCDC80 knockout mice exacerbated Ang II‐induced vascular expansion compared with CCDC80^fl/fl^ SM22α Cre^−^ mice (Figure 3D–F).

Histopathological analysis revealed typical AD lesions and determined that AD was the main cause of aortic rupture in CCDC80^fl/fl^ SM22α Cre^+^ mice receiving chronic Ang II treatment (Figure 3G,H). EVG staining and Masson's staining of aortic sections obtained from Ang II‐injected CCDC80^fl/fl^ SM22α Cre^+^ mice showed more severe collagen deposition and media degeneration, including elastic fiber fragmentation and disorganization (Figure 3G,H). These findings suggest that the deletion of VSMC‐specific CCDC80 exacerbates Ang II‐induced AD and that VSMC‐specific CCDC80 plays a major role in regulating vascular homeostasis.

### 2.4 CCDC80 Deficiency Accelerates Ang II‐Induced Contractile‐to‐Synthetic Phenotype Switching in VSMCs

Because CCDC80^−/−^ mice suddenly died from aortic rupture in the early days after Ang II administration, we harvested the aortas after 3 d of Ang II administration to evaluate the initial events before aortic rupture (Figure S6A,B, Supporting Information). Compared with WT mice, elastic fibers in the ASC and AA of CCDC80^−/−^ mice were partially disrupted and degraded and red blood cells were present between the elastic laminas of the degraded aortic walls (Figure S6C, Supporting Information).

**Figure 4:**
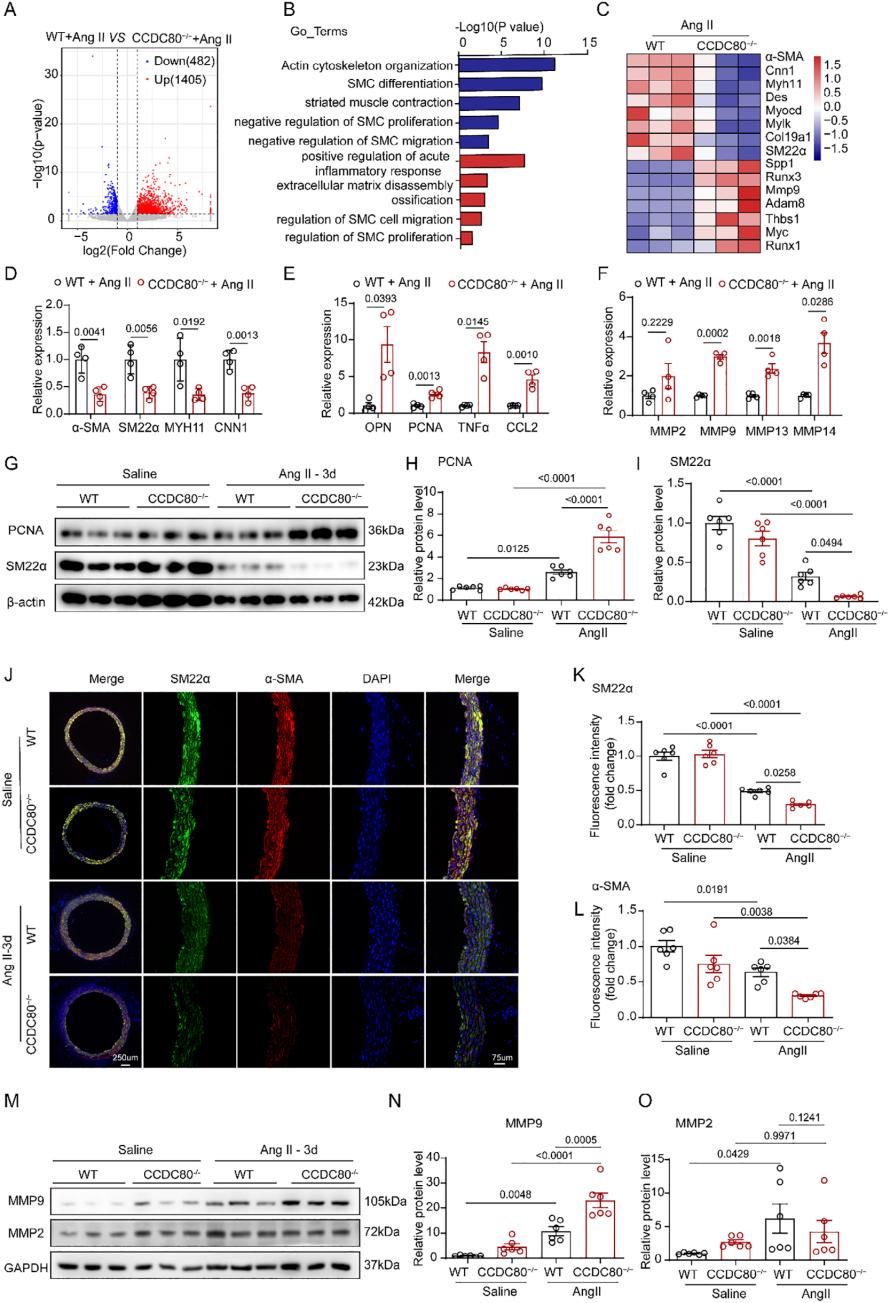
CCDC80 deletion facilitates Ang II‐induced contractile‐to‐synthetic phenotype switching in VSMCs. A) Volcano plot showing the genes of aortic tissues from WT and CCDC80^−/−^ mice after 3 d of Ang II administration. Upregulated genes are shown in red and downregulated genes are in blue. B) Gene Ontology enrichment analysis of differentially expressed genes (DEGs) of aortic tissues from WT and CCDC80^−/−^ mice after 3 d of Ang II administration. C) Heatmap of DEGs of aortic tissues from WT and CCDC80 KO mice after 3 d of Ang II administration. qPCR analysis of D) VSMC contractile, E) synthetic, inflammatory, and F) matrix metalloproteinase markers from RNA isolated from aortic tissues of WT and CCDC80^−/−^ mice, *n* = 4 per group. G–O) WT and CCDC80^−/−^ mice were treated with saline or Ang II for 3 d. G–I) Western blotting and quantification of SM22α and PCNA in aortic tissues, *n* = 6 per group. J–L) Immunofluorescence staining of SM22α (green) and α‐SMA (red) in ascending aorta (ASC). Nuclei were stained with DAPI (blue); scale bars = 250 µm and 75 µm. Quantification of α‐SMA and SM22α‐positive areas in the aortas, *n* = 6 per group. M–O) Western blotting and quantification of MMP2 and MMP9 in aortic tissues, *n* = 6 per group. Data are presented as mean ± SEM. Statistical analysis was performed using 2‐way ANOVA with Tukey's post hoc test for K, L, N, and O and Student's t‐test [D, E (PCNA and CCL2), F (MMP9 and MMP13)], Student's t‐test with Welch's correction [E (OPN and TNFα) and F(MMP2)], the Mann‐Whitney U test [F (MMP14)].

Next, mRNA sequencing was performed to identify differentially expressed genes (DEGs) in aortic tissues obtained from WT and CCDC80^−/−^ mice treated with Ang II for 3 d. In CCDC80^−/−^ samples, 1405 genes were upregulated (**Figure** **4**A), including those regulating ECM disassembly and SMC cell migration and proliferation (Figure 4B). By contrast, 482 genes (Figure 4A) were downregulated, particularly those enriched for SMC differentiation and muscle contraction (Figure 4B). Remarkably, the cell differentiation cluster was downregulated in CCDC80^−/−^ samples (Figure 4C), thereby indicating the activation of VSMC phenotype switching in CCDC80^−/−^ mice. In particular, the expression of VSMC contractile genes such as Myh11, SM22α, α‐SMA, and CNN1 was downregulated in CCDC80^−/−^ aortas, whereas the expression of VSMC synthetic genes such as Runx3, Thbs2, and Spp1 was upregulated (Figure 4C), thereby substantiating the crucial role of CCDC80 in VSMC phenotype switching in AD pathogenesis.

Synthetic VSMCs secrete MMPs and exhibit an inflammatory state via the expression of cell adhesion molecules and secretion of chemokines and inflammatory cytokines.^[^ ^24^ ^]^ To further confirm the abnormal VSMC phenotype switching in CCDC80^−/−^ mice, we performed qPCR on an array of VSMC‐related markers in aortic tissues harvested from CCDC80^−/−^ mice and their littermate controls after 3 d of Ang II administration. The absence of CCDC80 significantly reduced the contractile structural markers of VSMCs, such as α‐SMA, SM22α, CNN1, and Myh11 (Figure 4D). Moreover, an increased expression of synthetic genes, such as osteopontin, proliferating cell nuclear antigen (PCNA), tumor necrosis factor‐α (TNFα), CCL2, MMP9, MMP13, and MMP14, was observed in CCDC80 knockout mice compared with their littermate controls (Figure 4E,F). Furthermore, WB, IF staining, and IHC staining analyses revealed that Ang II treatment significantly downregulated the expression of contractile markers, including α‐SMA and SM22α, while upregulating synthetic markers such as PCNA in the aorta of both genotypes. Following Ang II administration, CCDC80^−/−^ mice exhibited a more pronounced reduction in contractile marker levels and a greater increase in synthetic marker expression in the aorta compared with WT mice (Figure 4G—L and Figure S7A–E, Supporting Information). Moreover, CCDC80 deficiency increased the expression of inflammatory factors (e.g., TNF‐α) but displayed no effect on VSMC apoptosis in the ASC (Figures S8 and S9, Supporting Information).

MMPs are a family of proteolytic enzymes that degrade ECM proteins and are critical for cell migration and tissue remodeling under physiological and pathological conditions.^[^ ^25^ ^]^ Studies have reported that MMP2 and MMP9 secreted by VSMCs play an important role in ECM degradation in aortic aneurysms and AD.^[^ ^26^, ^27^ ^]^ Following Ang II administration, MMP2/9 activity—determined via an in situ MMP activity assay—was enhanced in the aorta of CCDC80^−/−^ mice compared with WT mice (Figure S10A, B, Supporting Information). The aortas harvested from CCDC80^−/−^ mice expressed increased Ang II‐induced MMP9 mRNA compared with WT mice (Figure 4F). Furthermore, WB revealed that MMP2 and MMP9 expression were increased in Ang II‐induced WT mice. CCDC80^−/−^ mice exhibited a higher expression of Ang II‐induced aortic MMP9 expression compared with WT mice; however, Ang II‐induced aortic MMP2 expression was not significantly different between CCDC80^−/−^ and WT mice (Figure 4M–O). IF staining revealed that CCDC80 deficiency increased Ang II‐induced MMP9 expression in VSMCs (Figure S10C,D, Supporting Information). These data indicate that CCDC80 plays a novel protective role in the context of AD by regulating VSMC phenotype switching and decreasing MMP9 expression.

### 2.5 CCDC80 Deletion Accelerates Ang II‐Induced VSMC Phenotype Switching in Primary VSMCs In Vitro

**Figure 5:**
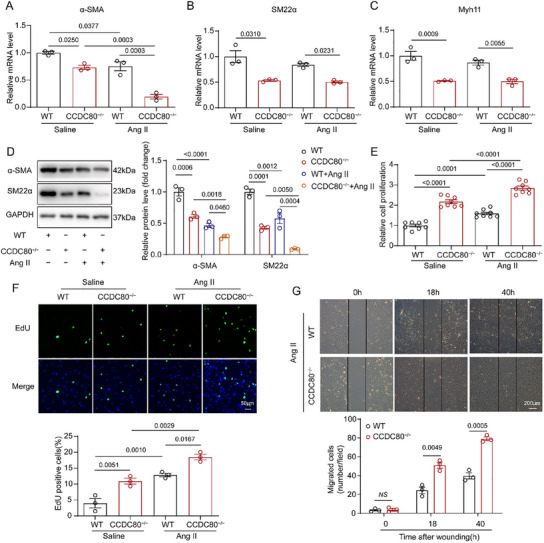
CCDC80 deletion accelerates Ang II‐induced phenotype switching in primary VSMCs in vitro. A–F) VSMCs isolated from wild‐type (WT) and CCDC80^−/−^ mice were serum‐starved for 24 h and treated with Ang II (1 µm). A–C) qPCR of α‐SMA, SM22α, and Myh11 mRNA in WT and CCDC80^−/−^ VSMCs after treatment with saline or Ang II (1 µm) for 24 h, *n* = 3 independent experiments. D) Western blotting of α‐SMA and SM22α in WT and CCDC80^−/−^ VSMCs following saline or Ang II (1 µm) treatment for 48 h, *n* = 3 independent experiments. E) Cell Counting Kit‐8 assay in WT and CCDC80^−/−^ VSMCs in response to Ang II (1 µm) treatment for 24 h, *n* = 8 independent experiments. F) EdU staining for cell proliferation in WT and CCDC80^−/−^ VSMCs after Ang II (1 µm) treatment for 24 h, *n* = 3 independent experiments. G) Monolayer cells were serum‐starved and scratched in the presence of Ang II (1 µm) to stimulate VSMC migration toward the wound. Representative images were photographed at 0, 18, and 40 h after scratching; migrated cells were quantified (number/field), *n* = 3 independent experiments. Data are presented as mean ± SEM. Statistical analysis was performed using one‐way ANOVA with Tukey's post hoc test for A–F and Student's t‐test for G. NS, statistically nonsignificant.

To investigate the effect of CCDC80 on the phenotype switching of VSMCs, we analyzed the expression of VSMC‐specific genes including α‐SMA, SM‐22α, and MYH11 using WB and qPCR. The expression of these genes was significantly downregulated in CCDC80^−/−^ VSMCs under quiescent and Ang II‐induced conditions (**Figure** **5**A–D). Moreover, cell proliferation experiments showed that VSMCs isolated from CCDC80^−/−^ mice exhibited greater proliferation after 24 h in culture, with or without Ang II treatment, compared with those from WT mice (Figure 5E,F). Next, we investigated to study the effect of CCDC80 deletion on VSMC migration and cell contraction capability using an in vitro scratch wound assay and collagen gel contractile assay. The results showed that Ang II‐induced cell migration was enhanced in CCDC80^−/−^ VSMCs (Figure 5G). And, Ang II‐induced VSMCs contraction was remarkably hampered in CCDC80^−/−^ VSMCs (Figure S11A,B, Supporting Information). These results further confirm that CCDC80 deletion facilitates VSMC phenotype switching from a quiescent contractile phenotype to an active synthetic phenotype.

### 2.6 CCDC80 Deficiency Activates the JAK2/STAT3 Pathway

To analyze the biological role of CCDC80 in AD, we studied CCDC80‐initiated transcriptional programs. Furthermore, we performed Kyoto Encyclopedia of Genes and Genomes (KEGG) pathway analysis of the genes differentially expressed between WT and CCDC80^−/−^ groups treated with Ang II. The results revealed that CCDC80 deficiency could regulate several pathways including the JAK/STAT, PI3K‐AKT, NF‐kappa B, Rap1, MAPK, and ECM‐receptor interaction signaling pathways (Figure S12A, Supporting Information). Consistently, gene‐set enrichment analysis revealed a positive correlation between CCDC80 deficiency and the JAK/STAT signaling pathway (NES = 1.4807, *p <* 0.0001; Figure S12B, Supporting Information).

**Figure 6:**
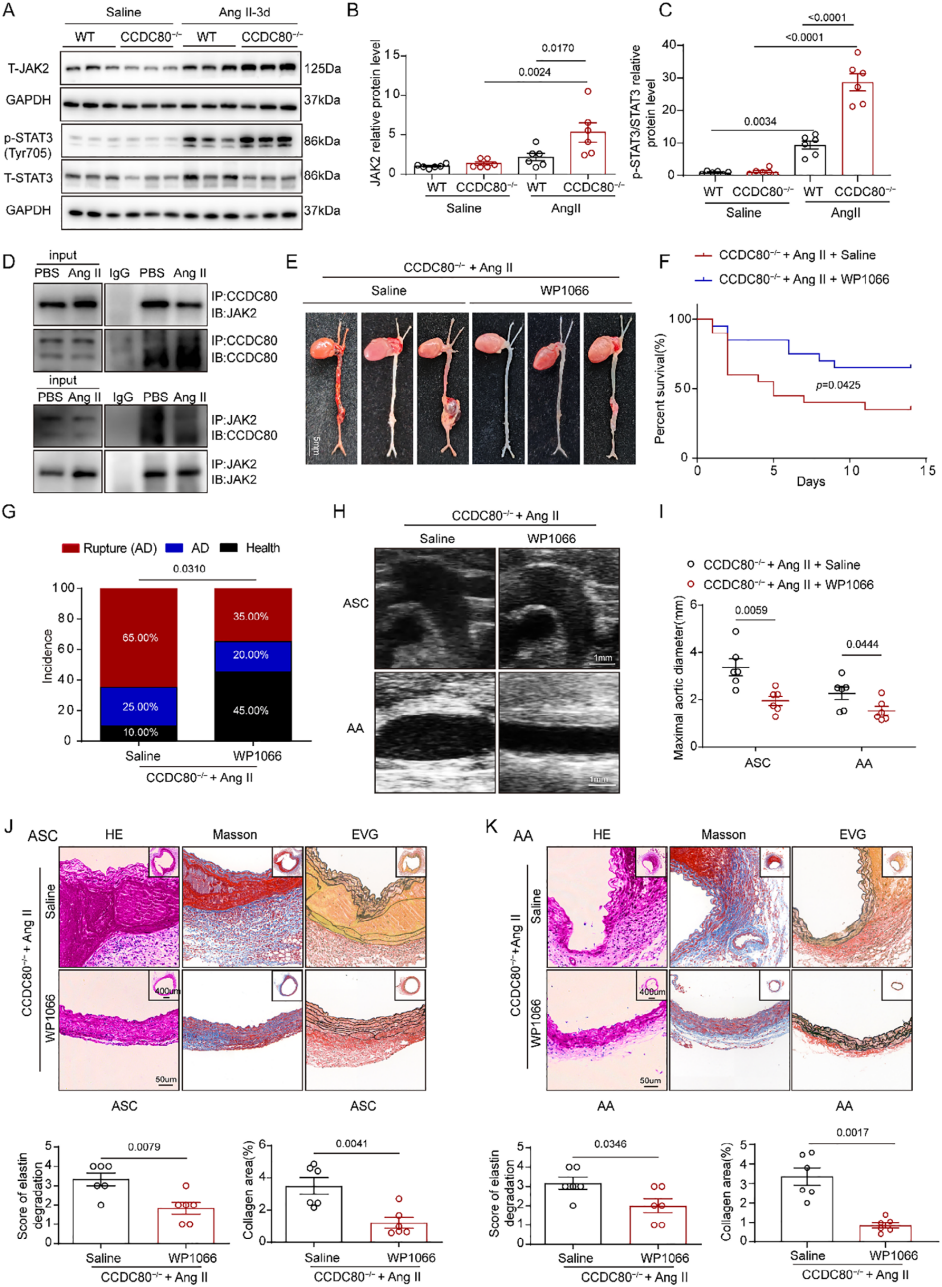
CCDC80 knockout activates the JAK2/STAT3 signaling pathway. A–C) Western blotting and quantification of JAK2, STAT3, and p‐STAT3 (Tyr705) in aortic tissues from WT and CCDC80^−/−^ mice after 3 d of Ang II administration, *n* = 6 per group. D) VSMCs isolated from WT mice were serum‐starved for 24 h and stimulated with Ang II (1 µm) for 48 h. Rabbit anti‐CCDC80 antibody was used to immunoprecipitate CCDC80 from the lysates of resting and Ang II‐stimulated VSMCs. Rabbit IgG was used as the negative control. Rabbit anti‐JAK2 antibody was used to immunoprecipitate JAK2 from the lysates of resting and Ang II‐stimulated VSMCs. Rabbit IgG was used as a negative control. E–K) CCDC80^−/−^ mice were treated with WP1066 or saline after Ang II administration for 14 d. E) Representative photographs of aortas from CCDC80^−/−^ mice treated with saline and WP1066 after Ang II administration for 14 d. (F) Kaplan–Meier survival curves for male CCDC80^−/−^ mice treated with saline and WP1066 during 14 d of Ang II administration, *n* = 20 per group. G) Quantification of incidence (AD, rupture, and health) in whole aortas, *n* = 20 per group. H) Representative ultrasound images of the ascending aorta (ASC) and abdominal aorta (AA); scale bar = 1 mm. I) Measurements of maximum ASC and AA, *n* = 6 per group. J,K) Representative transverse sections of J) ASC and K) AA from CCDC80^−/−^ mice stained with H&E, Masson's trichrome blue, and EVG. The grades of elastin degradation and collagen deposition in the aortic wall of mice were measured, *n* = 6 per group. Data are presented as mean ± SEM. Statistical analysis was performed using one‐way ANOVA with Tukey's post hoc test for (B,C), Student's t‐test [I–K (The grades of elastin degradation)], Student's t‐test with Welch's correction [K (collagen area)], the Kaplan–Meier method and log‐rank comparison tests for F, and A Fisher's exact test for G.

Previous studies have reported that the JAK/STAT signaling pathway is the central node for cytokine signaling and plays an important role in regulating cell proliferation, migration, and apoptosis as well as immunity, tissue repair, inflammation, and adipose differentiation.^[^ ^28^ ^]^ However, the abnormal activation of the JAK/STAT signaling pathway is significantly related to the occurrence of vascular diseases such as aortic aneurysm.^[^ ^29^ ^]^ Recent studies have reported that CCDC80 interacts with JAK2 (Janus kinase 2) through the DRO1‐URB‐DRS‐Equarin‐SRPUL/sushi repeat containing protein, x‐linked (DUDES) domain to regulate the downstream signal of STAT3 (Signal transducer and activator of transcription 3).^[^ ^30^ ^]^ Moreover, STAT3 can play an important role in cardiovascular diseases (such as atherosclerosis and pulmonary hypertension) through signal transduction and transcriptional activation.^[^ ^31^, ^32^ ^]^ Therefore, we further examined the role of the JAK2/STAT3 pathway in AD. CCDC80 deletion significantly promoted Ang II‐induced expression of JAK2 (**Figure** **6**A,B). Moreover, after 3 d of Ang II administration, the levels of phosphorylated STAT3 proteins, a downstream effector of JAK2, were significantly upregulated in CCDC80‐knockout aortas (Figure 6A,C). Furthermore, CCDC80 coimmunoprecipitated with JAK2 in mouse VSMCs and this interaction significantly decreased after Ang II stimulation (Figure 6D). These results suggest that CCDC80 strongly binds to JAK2 in the absence of Ang II and that CCDC80 deletion significantly activated the JAK2/STAT3 signaling pathway.

### 2.7 Pharmacological Blockage of the JAK2/STAT3 Pathway Protects CCDC80^−/−^ Mice from Ang II‐Induced AD

The JAK2/STAT3 pathway‐specific inhibitor WP1066 can irreversibly inhibit JAK2 and STAT3 by degrading JAK2 proteins and subsequently blocking downstream STAT3 pathway signal transduction and activation.^[^ ^33^ ^]^ To investigate the inhibitory effect of WP1066 on the JAK2/STAT3 pathway, we analyzed the expression of JAK2 and STAT3 using WB. The expression of JAK2 and p‐STAT3 was significantly downregulated in CCDC80^−/−^ mice aorta treated with WP1066 (Figure S13A,B, Supporting Information). Then, we examined the therapeutic effect of WP1066 on AD development in CCDC80^−/−^ mice with Ang II‐induced AD. Briefly, CCDC80^−/−^ mice were intraperitoneally injected with WP1066 daily (20 mg kg^−1^) following Ang II administration throughout the 2 weeks of modeling.

Notably, compared with CCDC80^−/−^ mice that did not receive WP1066 treatment, WP1066‐treated CCDC80^−/−^ mice demonstrated significantly lower AD formation (90.00% vs 55.00%, *p* < 0.05; Figure 6E,G) and lethality (Figure 6F,G) as well as mitigated aortic dilation (Figure 6H,I). Similarly, WP1066 treatment exerted no impact on blood pressure (Table S4, Supporting Information). Histological analyses via H&E, EVG, and Masson's staining techniques revealed that WP1066 ameliorated elastin disorganization, elastic fiber degradation, and collagen deposition (Figure 6J,K), thereby further confirming the critical role of the JAK2/STAT3 pathway in AD progression.

### 2.8 CCDC80 Maintains the Contractile Phenotype of VSMCs by Blockage of the JAK2/STAT3 Pathway

**Figure 7:**
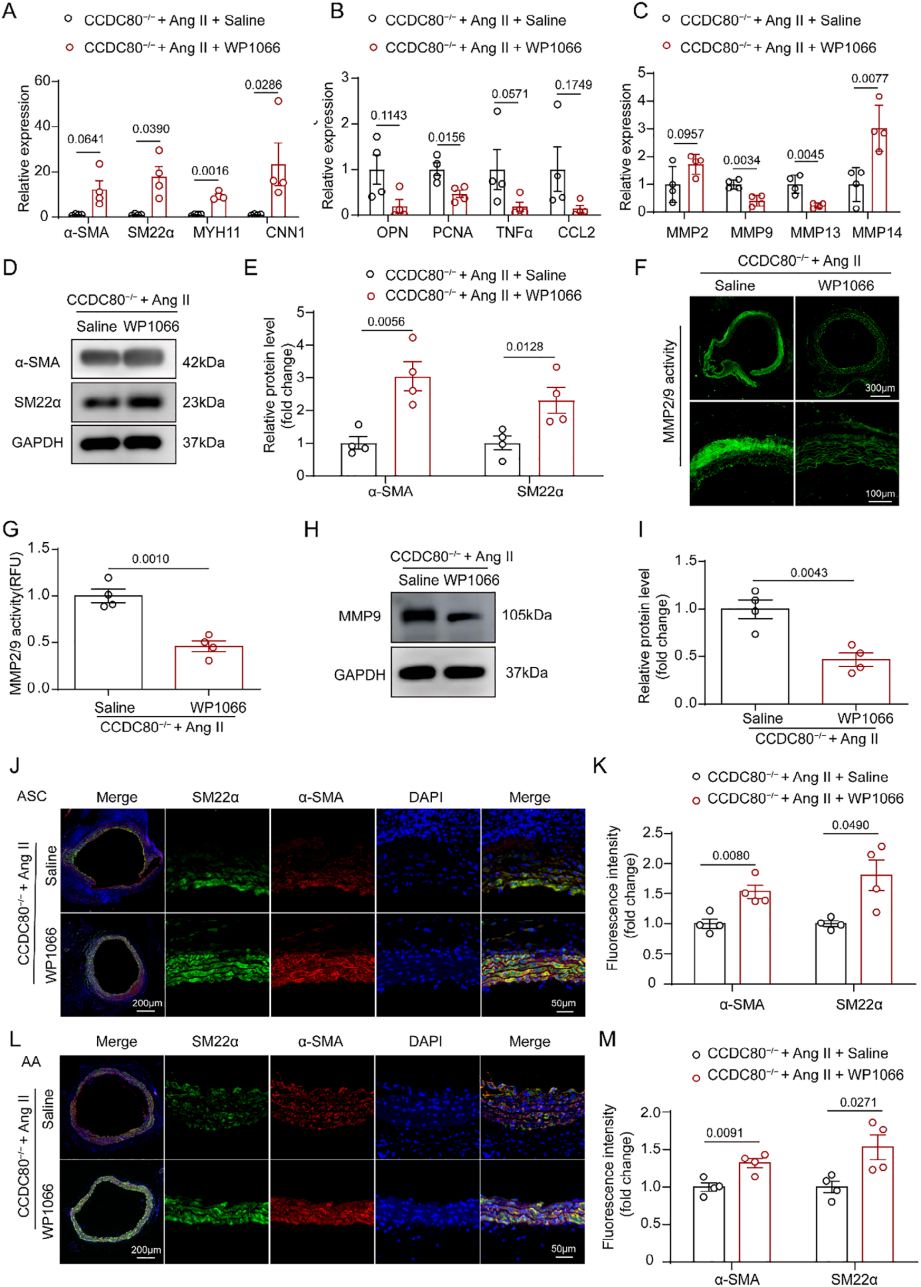
Blocking the JAK2/STAT3 pathway increases the contractile phenotypic marker of VSMCs in Ang II‐induced CCDC80^−/−^ mice. A–F) CCDC80^−/−^ mice were treated with WP1066 or saline after Ang II administration for 3 d. A–C) qPCR analysis of A) VSMC contractile, B) synthetic, inflammatory, and C) matrix metalloproteinase markers from RNA isolated from aortic tissues of CCDC80^−/−^ mice treated with WP1066 or saline after Ang II administration for 3 d, *n* = 4 per group. D,E) Western blotting and quantification of SM22α and α‐SMA in aortic tissues, *n* = 4 per group. F,G) In situ fluorescence zymography for MMP2/9 activity and quantification of MMP2/9‐positive area in aortas, *n* = 4 per group. H,I) Western blotting and quantification of MMP9 in indicated groups, *n* = 4 per group. J–M) Immunofluorescence staining of SM22α (green) and α‐SMA (red) in ascending aorta (ASC) and abdominal aorta (AA). Nuclei were stained with DAPI (blue); scale bars = 200 and 50 µm. Quantification of SM22α‐ and α‐SMA‐positive areas in aortas, *n* = 4 per group. Data are presented as mean ± SEM. Statistical analysis was performed using Student's t‐test [B (PCNA), C,E, K(α‐SMA) and M], Student's t‐test with Welch's correction [B (CCL2) and K(SM22α)], the Mann‐Whitney U test [A (CNN1), B (OPN and TNFα)].

To investigate the effect of WP1066 on phenotype switching, we performed qPCR on an array of VSMC‐related markers in aortic tissue harvested from CCDC80^−/−^ mice. Following Ang II administration, compared with mice that received no WP1066 treatment, WP1066‐treated CCDC80^−/−^ mice exhibited increased expression of VSMC contractile structural markers (including α‐SMA, SM22α, and Myh11; **Figure** **7**A), reduced expression of synthetic genes (including PCNA, MMP9, MMP13, and MMP14), and exerted no effect on CNN1, TNFα, CCL2, and MMP2 (Figure 7B,C). Similarly, WB and IF staining revealed that compared with the absence of WP1066 treatment, WP1066 administration resulted in a significant increase in contractile markers, such as α‐SMA and SM22α, in the aorta (Figure 7D,E,J–M). MMP9 expression and MMP activity were decreased in Ang II‐induced CCDC80^−/−^ mice treated with WP1066 (Figure 7F–I). These data suggest that JAK2/STAT3 is located downstream of CCDC80 and thus mediates VSMC differentiation and vascular degradation.

### 2.9 AD Triggers VSMC Phenotype Switching in the Human Aorta

**Figure 8:**
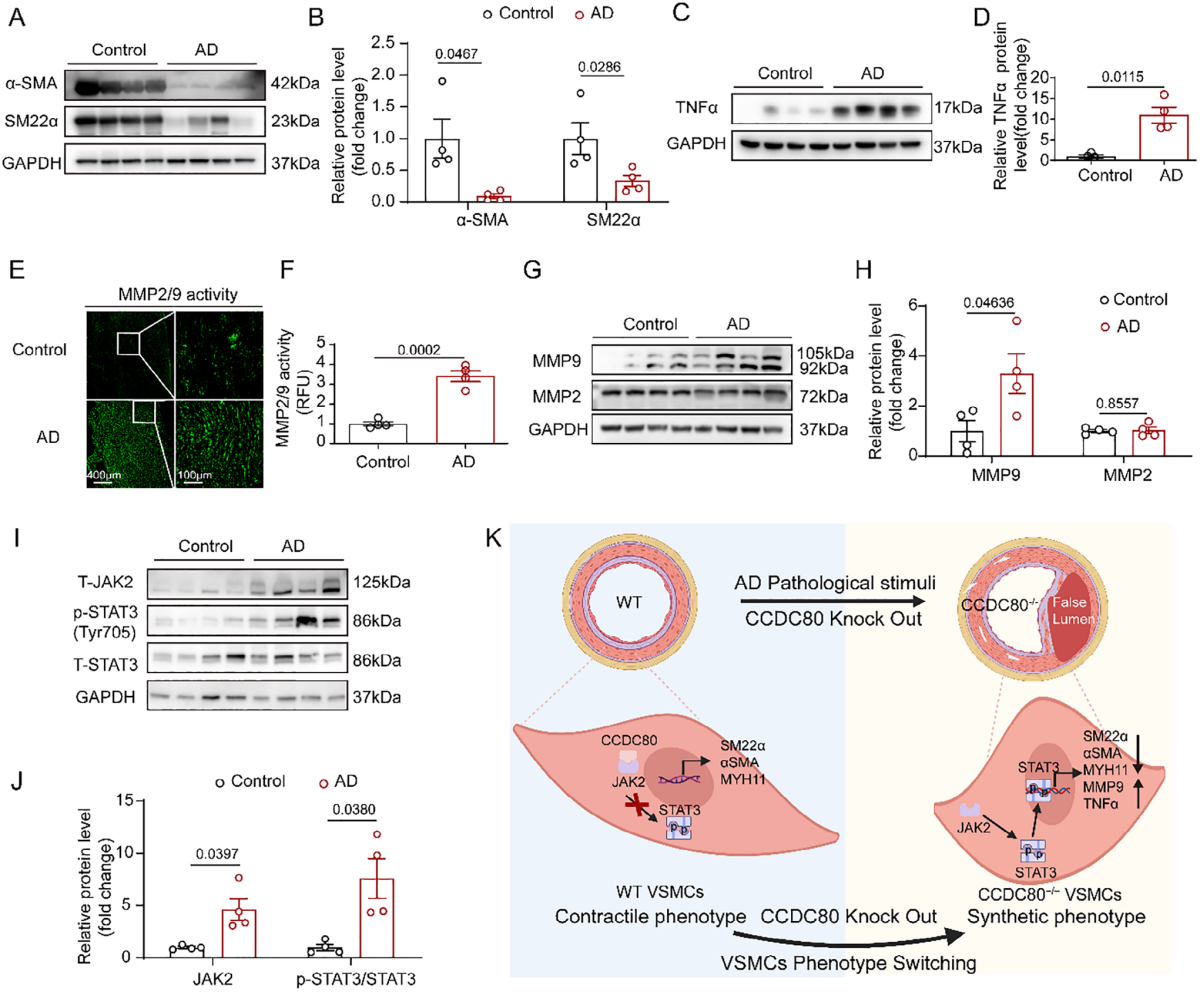
AD triggers VSMC phenotype switching in the human aorta. A,B) Western blotting and quantification of SM22α and α‐SMA in normal aorta and AD tissue, *n* = 4 per group. C,D) Western blotting and quantification of TNFα in normal aorta and AD tissue, *n* = 4 per group. E,F) In situ fluorescence zymography for MMP2/9 activity and quantification of MMP2/9‐positive areas in the aorta, *n* = 4 per group. G,H) Western blotting and quantification of MMP2 and MMP9 protein levels in normal aorta and AD tissue, *n* = 4 per group. I,J) Western blotting and quantification of JAK2, STAT3, and p‐STAT3 (Tyr705) protein levels in normal aorta and AD tissue, *n* = 4 per group. K) Graphic illustration of the role of VSMC‐specific CCDC80 deficiency in AD. VSMC‐specific CCDC80 deficiency accelerates the progression of AD by activating the JAK2/STAT3 pathway involved in regulating phenotype switching and function in VSMCs. AD, aortic dissection. Data are presented as mean ± SEM. Statistical analysis was performed using Student's t‐test [B(SM22α), F,H,J], Student's t‐test with D) Welch's correction, and the Mann‐Whitney U test [B(α‐SMA) and J].

The present study showed that CCDC80 could aggravate AD by promoting VSMC phenotype switching in mice. We investigated whether VSMC phenotype switching is exacerbated in human AD. Consistent with the results observed in mice AD, a decreased expression of α‐SMA and SM22α was observed in human AD compared with human normal aorta (**Figure** **8**A,B). Similarly, TNFα expression was elevated in the aorta of patients with AD compared with the aorta of normal individuals (Figure 8C,D). We assessed the MMP activity in vivo using an in situ zymography assay. AD increased MMP2/9 activity (Figure 8E,F). Furthermore, MMP9 expression increased in the aorta of patients with AD compared with that in the aorta of normal individuals; MMP2 expression remained unchanged (Figure 8G,H). In addition, JAK2 and phosphorylation levels of STAT3 proteins were upregulated in the aorta of patients with AD compared with that in the aorta of normal individuals (Figure 8I,J). These findings suggest that VSMC phenotype switching is involved in the formation and development of human AD.

## 3 Discussion

Accumulating evidence indicates that CCDC80 plays essential roles in the pathogenesis of various cardiovascular diseases, such as atherosclerosis, vascular remodeling, and myocardial hypertrophy.^[^ ^13^, ^15^, ^16^ ^]^ However, the role of CCDC80 in AD has not yet been reported. In the present study, human tissues, mouse knockout models, and in vitro approaches were used to identify the critical protective role played by CCDC80 for preventing AD. We observed that AD is accompanied by a decrease in CCDC80 in human and mice aortas and that the genetic ablation of CCDC80 caused an increase in the frequency and severity of AD in a murine AD model. CCDC80 could interact with JAK2 and its deficiency facilitated VSMC phenotype switching from a contractile to a synthetic phenotype by activating the JAK2/STAT3 signaling pathway. Furthermore, WP1066 decreased the formation and rupture of AD in CCDC80 knockout mice through mitigating VSMC phenotype switching. Collectively, to the best of our knowledge, these findings provide the first evidence to report a causal role for the loss of CCDC80 in promoting AD.

CCDC80 (also known as URB, SSG1, and DRO1), a member of the coiled‐coil domain‐containing protein family, regulates several biological processes such as tumor inhibition, adipocyte differentiation, and energy metabolism.^[^ ^34^, ^35^, ^36^, ^37^ ^]^ Emerging evidence has shown that CCDC80 plays a pivotal role in regulating cardiovascular remodeling and homeostasis.^[^ ^14^, ^15^, ^16^ ^]^ Although CCDC80 reportedly accelerates atherosclerosis by decreasing lipoprotein lipase expression,^[^ ^15^ ^]^ we unexpectedly found a protective effect of CCDC80 on AD development. This is the first study to identify that CCDC80 expression is markedly downregulated in both human and murine AD tissues, preferentially in VSMCs. We speculate that the decreased CCDC80 levels in AD may be an important for AD progression. To obtain direct evidence supporting the protective effect of CCDC80 in AD, a genetic loss‐of‐function approach was employed. The findings demonstrated that VSMC‐specific CCDC80 deficiency significantly accelerated AD formation in murine models. Collectively, these data demonstrate that VSMC‐derived CCDC80 may play an integral role in the pathogenesis of AD by regulating vascular integrity and function.

VSMCs are the main cell types in the arterial media and play an indispensable role in maintaining vascular structure and function.^[^ ^11^ ^]^ VSMCs are not terminally differentiated and retain extremely high phenotypic plasticity, thereby enabling them to regulate and respond to stress signals.^[^ ^38^ ^]^ Under pathological stimulation, VSMCs convert from a contractile to synthetic phenotype, thereby leading to adverse vascular remodeling and vascular malfunction.^[^ ^5^ ^]^ The synthetic phenotype is the basic element responsible for ECM degradation, aortic wall weakness, and aortic rupture.^[^ ^39^ ^]^ VSMC phenotype switching is associated with aortic aneurysm and AD. A deficiency in ALDH2, a member of the aldehyde dehydrogenase 2 family, inhibited VSMC phenotype switching in a miR‐31‐5p‐myocardin–dependent manner, which is associated with a lower risk of aortic aneurysm and AD in patients and mice.^[^ ^40^ ^]^ The XBP1u‐FoxO4‐myocardin axis plays a pivotal role in maintaining VSMC contractile phenotype and preventing aortic aneurysm formation.^[^ ^41^ ^]^ Macrophage‐derived Lgmn binds to integrin αvβ3 in VSMCs, thereby attenuating the activation of Rho GTPase, downregulating VSMC differentiation markers, and ultimately exacerbating the development of thoracic AD.^[^ ^42^ ^]^ Genetic ablation with Anxa1 downregulated the JunB/MYL9 pathway to promote the conversion of VSMCs to a synthetic phenotype; this contributed to accelerating the development or progression of acute AD.^[^ ^43^ ^]^ In the present study, CCDC80 deficiency reduced the expression of VSMC contractile markers and increased the levels of synthetic markers, thereby facilitating AD progression. Our findings are consistent with those of previous studies that VSMC phenotype switching is involved in aneurysm formation or AD. Our study provides novel insights in the field of VSMC phenotype switching in AD pathology.

Extensive ECM fragmentation is an important characteristic of AD. The ECM influences the behavior of vascular cells and provide structural support during physiological and pathological processes.^[^ ^44^ ^]^ Degradation of elastin in the ECM can promote aortic instability, thereby contributing to various vascular diseases.^[^ ^45^, ^46^ ^]^ MMPs are proteolytic enzymes that degrade ECM and induce cell migration and proliferation.^[^ ^25^ ^]^ Increased MMPs, particularly MMP‐2 and MMP‐9, degrade the ECM, thereby weakening the aortic wall and leading to the formation of aortic aneurysms and AD.^[^ ^26^ ^]^ In the present study, we noted that CCDC80 deficiency increased the expression of MMPs secreted by VSMCs, particularly that of MMP9, which promoted the degradation of elastin, thereby exacerbating the occurrence of AD. This further confirmed that synthetic VSMCs weakened the aortic wall and promoted aortic wall rupture by secreting MMP9.

The JAK2/STAT3 signaling pathway plays an important role in cytokine signaling. Pathological stimulation induces the tyrosine phosphorylation of JAK2 kinase and activation of STAT3.^[^ ^47^ ^]^ Activated STAT3 translocates to the nucleus where it combines with cis‐induced elements and promotes the activation of transcription of early growth response genes.^[^ ^48^ ^]^ Consistent with other studies, we observed that Ang II increased the expression of JAK2 and promoted STAT3 phosphorylation. Moreover, we found that CCDC80 deficiency significantly augmented STAT3 phosphorylation and JAK2 expression. These results suggest that CCDC80 plays a regulatory role in the JAK2/STAT3 signaling pathway. Previous studies have shown that CCDC80 binds to phosphorylated tyrosine JAK2 through its DUDES domain and subsequently activates STAT3 phosphorylation on Tyr705.^[^ ^30^ ^]^ In the present study, we observed that JAK2 interacted with CCDC80, whereas the interaction between JAK2 and CCDC80 was weakened after Ang II administration. This indicates that in the basal state, JAK2 protein binds to CCDC80 to inhibit the activation of the downstream STAT3 pathway; under pathological stimuli, JAK2 phosphorylation is enhanced, which further activates the STAT3 pathway, thereby leading to adverse vascular remodeling.

The JAK2/STAT3 pathway is involved in various biological processes, including inflammation and tissue damage and repair, and is critical in the formation and development of AD and aortic aneurysm. Activation of the JAK2/STAT3 pathway was observed in aortic aneurysm tissues.^[^ ^49^ ^]^ Previous studies have shown that the formation of an aortic aneurysm was at least partially dependent on Ang II‐mediated STAT3 activation in ApoE^−/−^ mice.^[^ ^29^ ^]^ A recent study found that metformin could inhibit the progression of Ang II‐induced mouse aortic aneurysms by reducing the activity of the STAT3 signaling pathway.^[^ ^50^, ^51^ ^]^ In addition, STAT3 inhibitors (S3I‐301) could reduce the incidence and severity of Ang II‐induced aortic aneurysm formation by decreasing MMP activity and M1/M2 macrophage ratio.^[^ ^52^ ^]^ In the current study, we observed that WP1066 promoted the transformation of VSMCs to a contractile phenotype without altering the vascular inflammation response, thereby reducing the formation and rupture of AD in CCDC80‐deficient mice. These data further suggest that the inhibition of the JAK2/STAT3 signaling pathway mitigates the progression of AD in CCDC80‐knockout mice involved in ameliorating VSMC phenotype switching.

The present study has several limitations. First, although our study mainly focuses on VSMC‐specific CCDC80 in AD progression, the role of CCDC80 in immune cells and vascular endothelial cells cannot be ruled out. Studies using CCDC80 overexpression mice must be conducted to validate the protective role of CCDC80 in regulating VSMC homeostasis in AD progression. In addition, it is controversial to study the specific expression of VSMC by Tagln‐Cre driver, and the research results of this strain still need to be carefully interpreted.^[^ ^53^ ^]^ Second, although CCDC80 was shown to affect VSMC function by regulating their phenotypic transformation, the role of CCDC80 as a secreted protein in regulating endothelial cells, macrophages, and fibroblasts in AD requires further research. Third, it is unknown whether patients with AD possess CCDC80 mutations. Finally, we noted that WP1066 could limit the development of AD. In the future, other inhibitors or genetic knockdown of JAK2/STAT3 in VSMCs are important to explore its role in CCDC80 regulation of AD, and clinical research must confirm if WP1066 can be used in a therapy to delay the progression of AD.

In summary, our data identify CCDC80 as a promising new candidate that maintains VSMC homeostasis and prevents AD development and progression (Figure 8K). Our findings may lead to a paradigm shift in pharmacological therapy for AD and offer a potential target for pharmacological therapy and management strategies for this disease.

## 4 Experimental Section

### Materials

Antibodies against β‐actin (4970), GAPDH (5174T), Phospho‐Stat3 (Tyr705) (9145), STAT3 (9139T), and JAK2 (3230) for WB were purchased from Cell Signaling Technology (Boston, MA, USA). Antibodies against α‐SMA (SC32251) for WB and immunostaining were purchased from Santa Cruz Biotechnology, Inc. (Dallas, TX, USA). Antibodies against SM22α (Ab14106) for WB and immunostaining were purchased from Abcam (Cambridge, UK). Normal mouse IgG (2729) used for immunostaining and as a negative control for co‐immunoprecipitation (Co‐IP) assay was obtained from Cell Signaling Technology. Antibodies against PCNA (10205‐2‐AP) for WB and immunostaining were purchased from Proteintech Group, Inc. (Wuhan, China). Antibodies against TNFα (GB11188), MMP9 (GB11132), MMP2 (GB11130), and CD31 (GB12063) for WB and immunostaining were purchased from Servicebio Group, Inc. (Wuhan, China). Antibodies against CCDC80 (PAJ901Ra01) for immunostaining, WB and Co‐IP assay were purchased from Cloud‐Clone Corp. (Wuhan, China). Antibodies against CCDC80 (AF3410) used for immunostaining were purchased from BD Biosciences (Franklin Lakes, NJ, USA). Antibodies against JAK2 (3230) for Co‐IP assay were purchased from Cell Signaling Technology (Boston, MA, USA). BAPN (A3134) and Ang II (A9525) were purchased from Sigma (St. Louis, MO, USA). WP1066 (HY‐15312) was purchased from MCE (Monmouth, NJ, USA).

### Human Tissue Analysis

Human samples were processed according to protocols approved by the Shanghai Jiao Tong University institutional review board (IS21014). Informed consent was obtained from each participant according to the Ethics Committee of Shanghai Chest Hospital, Shanghai Jiao Tong University School of Medicine. The study was carried out according to the criteria mentioned in the Declaration of Helsinki. Human AD tissue samples were obtained from six patients undergoing aortic root and ASC replacement; five control aortic tissues were obtained from age‐matched organ donors undergoing heart transplant surgery without AD, aneurysm, coarctation, or previous aortic repair. Characteristics of patients and controls are presented in Table S5 (Supporting Information).

### Animals

Experimental protocols were assessed and approved by the Animal Ethics Committee of Shanghai Chest Hospital [KS(Y)22333]. Animal experimental procedures were conducted in accordance with the guidelines of the National Institutes of Health Guidelines on the Care and Use of Laboratory Animals for scientific purposes. Global CCDC80 knockout mice (CCDC80^−/−^) in a C57BL/6J background were generated by the Shanghai Model Organisms Center and maintained by mating CCDC80^+/−^ male mice with CCDC80^+/−^ female mice. WT littermates were used as controls. Conditional VSMC‐specific CCDC80 knockout mice (CCDC80^fl/fl^ SM22α Cre) were generated by breeding CCDC80^fl/fl^ mice with *SM22α Cre* [B6.Cg‐Tg(SM22α‐Cre)1Her/J, #01 7491] mice from Jackson Laboratory. CCDC80^fl/fl^ littermates were used as controls. A 12 h light/dark cycle was used; the mice had ad libitum access to diet and water and were housed under a temperature of 24 °C and humidity of 40%.

### Construction of CCDC80^−/−^ Mice

CRISPR/Cas9 technology was used to induce genetic mutations using guide RNA (gRNA) and Cas9 nuclease, resulting in the coding shift of the CCDC80 reading frame or premature termination of translation. The CCDC80 transcript was obtained from the Ensemble database (ENSMUST00000 099498.9). The gRNA target sequences were designed using the website (crispor.tefor.net) and the sequences with the highest score were selected (see Table S6 for the sequence, Supporting Information). Then, CCDC80‐specific gRNAs were obtained via in vitro transcription. F0 generation mice were obtained by injecting Cas9 nuclease and gRNAs into fertilized eggs. F0 generation mice confirmed using PCR (see Table S6 for primer information, Supporting Information) were mated with WT C57BL/6J mice to obtain F1 generation heterozygous mice (CCDC80^+/−^). CCDC80^+/−^ mice were inbred to obtain CCDC80^−/−^ mice. The gRNA target sequences and primer information are provided in Table S6 (Supporting Information).

### Construction of CCDC80^fl/fl^ SM22α Cre Mice

The target sequence was identified according to the gene structure of CCDC80; the gRNA for this site was then designed according to the target sequence. Following activity detection, the active gRNA and Cas9 were transcribed into RNA in vitro. The homologous recombinant vector (donor vector) was constructed using In‐Fusion cloning. The vector contained a 3.1‐kb 5′ homologous arm, 0.8 kb flox region, and 3.1‐kb 3′ homologous arm. Cas9 mRNA, gRNA, and donor vector were microinjected into the fertilized eggs of C57BL/6J mice to obtain F0 mice (see Table S7 for sequence, Supporting Information). Based on the structural analysis of the CCDC80 gene, exon 3 of the CCDC80‐201 transcript was selected as the flox region. The translation of exon 3 initiated at 65.9% of the gene‐coding region. Knocking out the region would cause a frameshift mutation in the reading frame, thereby leading to the early termination of translation. F0 generation mice identified by PCR (see Table S7 for primer information, Supporting Information) were mated with WT C57BL/6J mice to obtain F1 generation heterozygous mice (CCDC80^flox/+^). CCDC80^flox/+^ mice were inbred to obtain CCDC80^fl/fl^ mice. Then, CCDC80^fl/fl^ mice were bred with hemizygous *SM22α Cre* [B6. Cg ‐Tg (SM22α‐Cre)1Her/J, #017491] mice (see Table S7 for primer information, Supporting Information) from Jackson Laboratory to obtain CCDC80^fl/fl^ SM22α Cre mice. SM22α‐cre transgenic hemizygous mice are viable, fertile, and normal‐sized without any significant physical or behavioral abnormalities.^[^ ^54^ ^]^ These transgenic mice express Cre recombinase under the control of a mouse smooth muscle protein 22‐α (SM22α) promoter. When hybridized with strains containing loxP site‐flanked sequence of interest, CRE‐mediated recombination resulted in the deletion of flanker sequences in vascular smooth muscle cells. Thus, Mice hemizygous for the SM22α‐cre transgene were used for breeding, and female breeders also carried SM22‐Cre. The gRNA target sequences and primer information are provided in Table S7 (Supporting Information).

### AD Model

Ang II‐ or Ang II + BAPN‐induced AD models were established as previously reported.^[^ ^55^, ^56^, ^57^ ^]^ Briefly, 5‐12 week old male knockout (CCDC80^−/−^ and CCDC80^fl/fl^ SM22α Cre) and WT mice were randomly divided into Ang II or saline treatment groups.

### Ang II + BAPN‐Induced AD Model

At 5 weeks of age, mice were anesthetized and implanted with osmotic pumps (Alzet 2004, Durect Corp., Cupertino, CA, USA) filled with Ang II (1000 ng/kg/min, Sigma) for 4 weeks. The mice were provided regular diets and BAPN (1 g/kg/day, Sigma) dissolved in water. BAPN (1 g/kg/day, Sigma) was dissolved in water and provided at the same time as Ang II administering and was maintained for 4 weeks.

### Ang II‐Induced AD Model

At approximately 8–12 weeks of age, mice were anesthetized with isoflurane (2%) and implanted with osmotic pumps (Alzet 2002, Durect Corp.) filled with Ang II (1000 ng/kg/min, Sigma) for 2 weeks.

### WP1066 Treatment

Male CCDC80^−/−^ mice (aged 8–12 weeks) were administered WP1066 (20 mg kg^−1^ per d, MCE) or saline via an intraperitoneal injection for 2 weeks.

### Blood Pressure Measurements

Blood pressure was noninvasively measured in conscious mice using the tail‐cuff plethysmography method (BP‐2010A, Softron Biotechnology, Beijing, China). Mice were trained for the measurements before measuring the pressure levels. All measurements were performed at the same time (between 14 p.m. and 17 p.m.) to ensure accurate measurements and to avoid the influence of the circadian cycle. One set of six measurements was obtained for each animal; the average blood pressure was calculated.

### Tissue Collection and Analysis

At 2 weeks after the experiment, the mice were euthanized under deep anesthesia for blood collection. Then, the mice were perfused with saline, and aortic tissue was obtained and photographed. The maximum diameters of the ASC and AA were measured using calipers. Subsequently, the ASC and AA were sectioned and filled with an optical cutting temperature (OCT) compound or paraffin. The sectioned specimens (5 µm) were stained with H&E (Sigma), EVG (Abcam), and Masson's trichrome (Sigma). Other segments of the aorta were stored at −80 °C for molecular analysis.

### Echocardiographic Imaging

To evaluate the morphological characteristics of the thoracic aortas and AAs in vivo, ultrasound imaging was performed using a Vevo2100 cardiovascular ultrasound system (VisualSonics Inc., Ontario, Canada) with a 30 MHz transducer for mice. Following skin hair removal at ultrasonic sites, mice were anesthetized with 1.0%–2.0% isoflurane inhalation and gently fixed to a heated platform in supine position. By adjusting the direction of the ultrasonic probe, the diameters of the thoracic aorta and AA were detected in B mode.

### Mouse Aorta VSMCs

Mouse aorta VSMCs were isolated from the thoracic aortas of WT and CCDC80^−/−^ mice as described previously.^[^ ^58^ ^]^ Briefly, mouse aortic tissues were isolated and rinsed in phosphate‐buffered saline (PBS). Following the removal of the endothelium and adventitia, the tissues were cut into approximately 2 mm sections and treated with 0.2% collagenase type II (Worthington Biochemical Corporation, OH, USA, LS004176,) at 37 °C for 2 h. VSMCs were then cultured in Dulbecco's modified Eagle's medium/F12 supplemented with 10% fetal bovine serum and 1% penicillin‐streptomycin. The VSMCs were used in the experiments between passages 3 and 6. And, the VSMCs were serum‐starved for 24 h before drug treatment. Then, immunofluorescence staining was performed to detect the presence of α‐SMA to validate VSMCs (Figure S14, Supporting Information).

### Western Blotting

Protein lysates from aortic tissues or cells were lysed using lysis buffer (Roche, Mannheim, Germany, 4719964001) containing protease and phosphatase inhibitors (Roche). Protein concentrations were evaluated using the Bradford protein assay (Thermo Fisher Scientific, Waltham, MA, USA). Protein samples (20 µg per lane) were electrophoresed on sodium dodecyl sulfate–polyacrylamide gels and transferred to polyvinyl difluoride membranes. Membranes were blocked using 5% non‐fat dry milk for 1 h at room temperature and incubated with primary antibodies for 12 h at 4 °C. The membranes were washed and incubated for 1 h at room temperature with anti‐rabbit or anti‐mouse secondary antibodies conjugated to horseradish peroxidase (HRP). GAPDH or β‐actin acted as the control. The protein bands were detected via enhanced chemiluminescence (Bio‐Rad Laboratories, Inc., Hercules, CA, USA) and analyzed using the ImageJ software (National Institutes of Health, Bethesda, MD, USA).

### Real‐Time qPCR (RT‐qPCR)

Total RNA was extracted using TRIzol reagent (Invitrogen, Waltham, MA, USA) according to the manufacturer's protocol and reverse transcribed into cDNA using the PrimeScript RT Master Mix kit (Takara). Quantification of mRNA was performed using TB Green Premix Ex Taq (Takara); real‐time PCR experiments were conducted using the ABI PRISM 7300HT sequence detection system (Applied Biosystems, Waltham, MA, USA). The mRNA levels were normalized against GAPDH. The primer sequences used for q‐PCR are listed in Table S8 (Supporting Information).

### RNA Sequencing

Total RNA from the aortas was extracted using TRIzol reagent (Invitrogen, Waltham, MA, USA) following the manufacturer's protocol. The concentration, quality, and integrity of RNA was verified using the NanoDrop spectrophotometer (Thermo Fisher Scientific). RNA sequencing was performed on Novaseq 6000 (Illumina, San Diego, CA, USA). After obtaining data from the machine, it was converted to the original BCL file using real‐time analysis (version v3.4.4), split using the bcl2fastq (btq) software (version v2.19), and converted into FQ. The Fastp software (version 0.19.7) was used for quality control, involving the parameter fastp‐G‐Q5‐U50‐N 15‐L150. The DESeq21 R package was used to identify DEGs and corrected via the Benjamini–Hochberg method. A DEG is defined as one with an adjusted *p*‐value of <0.05 and |log2 (fold change) | of ≥1. A heatmap was generated using the pheatmap R package. KEGG and Gene ontology analyses were performed to assess pathway enrichment.

### Co‐IP Assay

VSMCs were stimulated with Ang II (1 µm) for 48 h and lysed in cold lysis buffer (87787; Thermo Fisher Scientific) with a protease inhibitor cocktail on ice for 30 min. The lysates were centrifuged at 12 000 g and 4 °C for 10 min. The cell lysates were centrifuged; protein concentrations were determined using the Pierce BCA Protein Assay kit and adjusted to ≈5 µg µL^−1^. Approximately 300 µg of the lysates were incubated with 15 µL of Protein A/G magnetic beads (88802; Thermo Fisher Scientific) at 4 °C overnight. Next, protein‐magnetic beads were incubated with rabbit anti‐JAK2 antibody, rabbit anti‐CCDC80 antibody, or anti‐IgG (2729; Cell Signaling Technology) at 4 °C overnight. Beads containing protein–antibody complexes were mixed with 2× loading buffer, boiled for 10 min, and analyzed with anti‐JAK2 and anti‐CCDC80 antibodies.

### Histological Analysis, IF Staining, and IHC Staining

AAs and ASCs were collected and fixed with 4% paraformaldehyde for 48 h and embedded in paraffin. Aortic tissue sections (5 µm) were stained with H&E to observe general morphology according to the manufacturer's protocol. EVG staining was performed to assess elastic fiber integrity; Masson's trichrome staining was performed to evaluate collagen content. Elastin degradation was identified as a discontinuation and widening of the elastic lamina and was evaluated by counting the number of breaks per vessel.^[^ ^59^ ^]^ The grade was defined as follows: Grade 1, no degradation; Grade 2, slightly degraded elastin; Grade 3, severe degradation of elastin, grade 4, Aortic rupture. Images were viewed using a microscope (Thermo Fisher Scientific, Waltham, MA, USA) and analyzed using the Image‐Pro Plus 6.0 software (Media Cybernetics Inc., Rockville, MD, USA) to assess the collagen content.

For IF staining, the formaldehyde‐fixed sections were permeabilized with 0.3% Triton, blocked with 5% bovine serum albumin, and incubated overnight with primary antibodies against α‐SMA (1:200, Santa Cruz Biotechnology), SM22α (1:200, Abcam), TNFα (1:200, Servicebio Group, Inc.), and CCDC80 (1:100, BD Biosciences) after continuous dewaxing, rehydration, and antigen repair. Then, the sections were incubated with green fluorescent secondary antibodies (Donkey Anti‐Rabbit IgG, 488 nm, Invitrogen), red fluorescent antibodies (Donkey Anti‐Mouse IgG, 555 nm, Invitrogen), or magenta fluorescent antibodies (Goat Anti‐rat IgG, 647 nm, Invitrogen). The nuclei were stained with 4′,6‐diamidino‐2‐phenylindole (DAPI, Beyotime, Shanghai, China) for 10 min. A fluorescence microscope was used for visualization (Leica, Germany).

For IHC staining, paraffin‐embedded sections (thickness of 5 µm) were treated with 3% hydrogen peroxide after continuous dewaxing, rehydration, and antigen repair. The sections were blocked with 5% normal fetal bovine serum, incubated with primary antibodies against CCDC80 (1:100, BD Biosciences) and PCNA (1:100, Proteintech Group, Inc.) overnight at 4 °C, and incubated with HRP‐conjugated secondary antibodies for 1 h. Blotting results were visualized with diaminobenzidine‐based HRP staining. Nuclei were counterstained with hematoxylin. The sections were imaged using fluorescence microscopy.

### In Situ Zymography

MMP2/9 activity was assessed using a Gelatinase Assay Kit (GMS80062.1, GenMed Scientifics Inc., Wilmington, DE, USA) according to the manufacturer's instructions. Briefly, unfixed ASCs or AAs were embedded with OCT and cut into 8‐µm‐thick serial sections. Fluorescein‐conjugated gelatin as a fluorogenic substrate was used to analyze gelatinolytic activity. Aortic tissue sections were incubated at 4 °C for 10 min and then maintained at 37 °C for 2 h. Fluorescein‐5‐isothiocyanate fluorescence was examined by fluorescence microscopy (Leica DM3000B, Leica Microsystems, Wetzlar, Germany) and determined using the ImageJ software.

### Cell Proliferation Assay (CCK‐8 Assay and EdU)

For cell counts, primary VSMCs isolated from WT and CCDC80^−/−^ mice were plated on 96‐well plates at a density of 1 × 10^4^ cells per well. To render the VSMCs quiescent, the VSMCs were starved in serum‐free medium for 24 h. Then, they were treated with Ang II (1 µm) for 48 h. Next, 10 µL of CCK‐8 reagent (Beyotime) was added to each well and the plates were incubated for 4 h. Absorbance values were measured at 450 nm using an enzyme standard instrument (Thermo Fisher Scientific).

The proliferation of VSMCs was detected using the BeyoClick EdU‐488 kit (Beyotime) according to the manufacturer's protocol. VSMCs were seeded at a density of 5.0 × 10^4^ cells in 24‐well plates and treated with Ang II (1 µm) for 48 h. Then, the VSMCs were incubated with 10 µmol L^−1^ EdU solution for 2 h at 37 °C and fixed with 4% PFA at 4 °C for 15 min. Finally, 100 µL click additive solution was added to the cells, following which nucleus staining with Hoechst 33342 was performed. The fluorescence signal was visualized using a fluorescence microscope.

### Scratch Wound Assay

Primary mouse WT and CCDC80^−/−^ VSMCs were isolated and seeded in six‐well plates. Straight‐line scratches were made on a monolayer of VSMCs using a 200 µL sterile pipette tip. The cells were washed with PBS and treated with or without Ang II (1 µm) containing normal growth medium for an additional 18 and 40 h. The number of VSMCs that migrated into the wound was visualized using a microscope (Thermo Fisher Scientific, Waltham, MA, USA).

### Collagen Gel Contraction Assay

The contractile capabilities of VSMCs were evaluated using a collagen gel‐based assay kit (Cell Biolabs, Cell contraction assay, Cat No. CBA‐201). WT and CCDC 80 ^−/−^ VSMCs were cultured in serum‐free medium for 24 h before seeding them into collagen gels. VSMCs were then harvested and resuspended in complete media at 2 × 10^6^ cells mL^−1^. Collagen gels were prepared as one part of VSMC suspension and four parts cold Collagen Gel Working Solution. A 500 µL volume of the cell–collagen mixture was added to each well of the 24‐well plate, which was then incubated for 1 h at 37 °C. Polymerized gels were added as an additional 1 mL of complete media with Ang II (1 µm) and further incubated for 2 d at 37 °C to develop contractile stress. Then, the collagen gels were gently released using a pipette tip. Digital photographs of the collagen gel lattices were taken after 12 and 24 h, and the surface area of the gel was measured using Image J.

### Terminal Deoxynucleotidyltransferase‐Mediated dUTP Nick‐End Labeling (TUNEL) Assay

TUNEL staining was performed to detect cell apoptosis using the In Situ Cell Death Detection kit (Beyotime). Briefly, aortic tissue sections were fixed in 4% paraformaldehyde for 15 min and permeabilized with 0.25% Triton X‐100 for 10 min. Then, tissue samples were incubated with TUNEL reagent at 37 °C for 50 min. The nuclei were stained with DAPI for 10 min. Tissue sections were visualized using a fluorescence microscope (Leica, Germany).

### Statistical Analysis

Continuous data were expressed as the mean ± standard error of the mean (SEM), categorical variables were shown as the percentage. The data distribution normality was determined by the Shapiro‐Wilk test, and the equality of variances by F test. For comparisons between two‐group of normally distributed data, Student's t test was used to assess similar variances, or Welch's correction was used to assess unequal variances. The Mann‐Whitney U test was performed for variables not normally distributed. For multiple comparisons (more than two groups), the homogeneity of variance was assessed by the Brown‐Forsythe test. Comparisons were performed by a 1‐way and 2‐way ANOVA analysis followed by Tukey post hoc multiple comparisons test (equal variances) or a Welch ANOVA test followed by a post hoc analysis using the Tamhane T2 method (unequal variances). For data with nonnormally distributed variables, Kruskal‐Wallis test with Dunn's multiple comparisons test was used for multigroup comparisons. The survival curves were assessed using the Kaplan–Meier method and compared using log‐rank tests. A Fisher's exact test was used to compare bivariate categorical variables. The result was statistically significant if the *p*‐value was <0.05. Statistical analysis was performed using GraphPad Prism version 8.0 software.

## Conflict of Interest

The authors declare no conflict of interest.

## Author Contributions

Q.Q.X., Y.L., and B.C. contributed equally to this work. L.H.S. and B.H. conceived and designed the research. Q.Q.X., Y.L., and B.C. performed experiments. Q.Q.X., X.Y.H., L.F., F.L., L.C., K.X., W.F.Z., X.L.W., and A.W.Y analyzed data. Q.Q.X. and Y.L. wrote the manuscript. X.W., Z.H.C., F.Z., Q.S., B.Z., L.H.S., and B.H. edited the manuscript. All authors approved the submitted and final versions.

## Supporting information



Supporting Information

## Data Availability

The data that support the findings of this study are available from the corresponding author upon reasonable request.
