# Supplementary material for: CCDC80 Protects against Aortic Dissection and Rupture by Maintaining the Contractile Smooth Muscle Cell Phenotype
Source: Adv Sci (Weinh). 2025 Apr 25;12(26):2502108. doi: 10.1002/advs.202502108 (PMC12245012; doi:10.1002/advs.202502108)
Supplement: Supplementary file 1 — Supporting Information [file ADVS-12-2502108-s001.docx]

**Supporting Information**

**CCDC80 Protects against Aortic Dissection and Rupture by Maintaining the** **Contractile Smooth Muscle Cell Phenotype**

Qingqing Xiao, Yi Li, Bin Cai, Xiying Huang, Liang Fang, Feng Liang, Long Chen, Ke Xu, Weifeng Zhang, Xiaolei Wang, Anwen Yin, Xia Wang, Zhaohua Cai, Fei Zhuang, Qin Shao, Bin Zhou, Berthold Hocher, Ben He^*^, Linghong Shen^*^

Q. Xiao, Y. Li, X. Huang, F. Liang, L. Chen, K. Xu, W. Zhang, X. Wang, A. Yin, X. Wang, Z. Cai, F. Zhuang, Q. Shao, B. He, L. Shen

Department of Cardiology

Shanghai Chest Hospital

Shanghai Jiao Tong University School of Medicine

Shanghai 200030, China.

E-mail: [shenlinghong@sjtu.edu.cn](mailto:shenlinghong@sjtu.edu.cn); [heben241@126.com](mailto:heben241@126.com).

Q. Xiao

Department of Cardiology

Shanghai General Hospital

Shanghai Jiao Tong University School of Medicine

Shanghai 200080, China.

B. Cai

Department of Rheumatology

Peking Union Medical College Hospital

Peking Union Medical College & Chinese Academy of Medical Sciences.

Beijing 100730, China.

L. Fang

Department of Cardiac Surgery

Shanghai Chest Hospital

Shanghai Jiaotong University School of Medicine

Shanghai 200030, China.

B. Zhou

Chinese Academy of Sciences

University of Chinese Academy of Sciences

Shanghai 200032, China

B. Hocher

Fifth Department of Medicine (Nephrology/Endocrinology/Rheumatology/Pneumology)

University Medical Centre Mannheim

University of Heidelberg

Heidelberg 69123, Germany.

Reproductive and Genetic Hospital of CITIC-Xiangya

People’s Republic of China

Changsha 410028, China

IMD Institut fur Medizinische Diagnostik Berlin-Potsdam GbR

Berlin 14473, Germany

**Supplemental Figures and Tables**

**Table S1. Blood pressure in CCDC80^−/−^ mice and their WT littermates injected with saline or Ang II + BAPN**

|  | Saline | |  | Ang II+BAPN | |
| --- | --- | --- | --- | --- | --- |
|  | WT | CCDC80^−/−^ | WT | | CCDC80^−/−^ |
| SBP (mmHg) | 110 ± 2 | 110 ± 4 |  | 146 ± 5^***^ | 145 ± 7^##^ |
| DBP (mmHg) | 79 ± 2 | 70 ± 5 |  | 102 ± 6^**^ | 106 ± 7^##^ |
| MBP (mmHg) | 89 ± 2 | 83 ± 4 |  | 117 ± 5^***^ | 119.0 ± 7^##^ |

Data are presented as mean ± SEM, n = 6 in each group. ***p* < 0.01, ****p* < 0.001 *vs.* saline-injected WT mice. ^##^*p* < 0.01 *vs.* saline-injected CCDC80^−/−^ mice. Abbreviations: Ang II, angiotensin II; SBP, systolic blood pressure; DBP, diastolic blood pressure; MBP, mean blood pressure.

**Table S2. Blood pressure in CCDC80^−/−^ mice and their WT littermates injected with saline or Ang II**

|  | Saline | |  | Ang II | |
| --- | --- | --- | --- | --- | --- |
|  | WT | CCDC80^−/−^ | WT | | CCDC80^−/−^ |
| SBP (mmHg) | 97 ± 1.7 | 101 ± 2 |  | 143 ± 4^***^ | 147 ± 5^###^ |
| DBP (mmHg) | 70 ± 3 | 69 ± 2 |  | 97 ± 5^***^ | 85 ± 7^###^ |
| MBP (mmHg) | 79 ± 2 | 80± 2 |  | 112 ± 3^***^ | 106 ± 6^###^ |

Data are presented as mean ± SEM, n = 8 in each group. ****p* < 0.001 *vs.* saline-injected WT mice. ^###^*p* < 0.001 *vs.* saline-injected CCDC80^−/−^ mice. Abbreviations: Ang II, angiotensin II; SBP, systolic blood pressure; DBP, diastolic blood pressure; MBP, mean blood pressure.

**Table S3. Blood pressure in CCDC80^fl/fl^ SM22α Cre^+^ mice and their CCDC80^fl/fl^ SM22α Cre^−^ littermates were injected with saline or Ang II**

|  | Saline | |  | Ang II | | | |
| --- | --- | --- | --- | --- | --- | --- | --- |
|  | CCDC80^fl/fl^ SM22αCre^−^ | CCDC80^fl/fl^  SM22αCre^+^ | CCDC80^fl/fl^ SM22αCre^−^ | | | CCDC80^fl/fl^  SM22αCre^+^ |  |
| SBP (mmHg) | 115 ± 2 | 111 ± 8 |  | | 158 ± 10^**^ | 159 ±7^##^ |  |
| DBP (mmHg) | 73 ± 1 | 61 ± 10 |  | | 91 ± 5^*^ | 91 ± 5^#^ |  |
| MBP (mmHg) | 86 ± 1 | 78 ± 9 |  | | 114 ± 6^**^ | 120 ± 5^##^ |  |

Data are presented as mean ± SEM, n = 5 in each group. **p* < 0.05, ***p* < 0.01****p* < 0.001 *vs.* saline-treated WT mice. ^#^*p* < 0.05, ^##^*p* < 0.01 *vs.* saline-treated CCDC80^−/−^ mice. Abbreviations: Ang II, angiotensin II; SBP, systolic blood pressure; DBP, diastolic blood pressure; MBP, mean blood pressure.

**Table S4. Blood pressure in CCDC80^−/−^ mice injected with saline or WP1066 following Ang II administration**

|  | CCDC80^−/−^+ Ang II+Saline |  | CCDC80^−/−^+ Ang II+WP1066 |
| --- | --- | --- | --- |
| SBP (mmHg) | 135 ± 3 |  | 135 ± 6 |
| DBP (mmHg) | 88 ± 5 |  | 83 ± 6 |
| MBP (mmHg) | 104 ± 4 |  | 101 ± 6 |

Data are presented as mean ± SEM, n = 5 in each group. Abbreviations: Ang II, angiotensin II; SBP, systolic blood pressure; DBP, diastolic blood pressure; MBP, mean blood pressure.

**Table S5. Patient characteristics**

|  | AD  n=6 | Control  n=5 | *p* value |
| --- | --- | --- | --- |
| Male (%) | 5(83.33%) | 2(40.00%) | 0.1368 |
| Age (mean±SD) | 56.5000 ± 5.1820 | 48.2000 ± 7.7680 | 0.3832 |
| Hypertension (%)) | 4 (66.67%) | 2(40.00%) | 0.3765 |
| Smoking (%) | 4 (66.67%) | 1(20.00%) | 0.1217 |
| Diabetes (%) | 0(0.00%) | 2(40.00%) | 0.0868 |
| Hyperlipidemia (%) | 3 (50.00%) | 3 (60.00%) | 0.7401 |
| Coronary heart disease | 0 (0.00%) | 0 (0.00%) | 1.0000 |
| Total cholesterol  (mmol/L, mean±SD) | 4.7930 ± 0.3620 | 4.4500 ± 0.5483 | 0.6027 |
| TG (mmol/L, mean±SD) | 2.2720 ± 1.0630 | 1.5620 ± 0.2583 | 0.5680 |
| LDL (mmol/L, mean±SD) | 2.5470 ± 0.5068 | 2.9520 ± 0.4916 | 0.5845 |
| HDL (mmol/L, mean±SD) | 1.4980 ± 0.2742 | 1.0500 ± 0.0816 | 0.1845 |

Data are shown as a number (percent) or as the mean ± standard error of the mean (SEM). AD: ascending thoracic aortic tissue from patients with acute ascending thoracic aortic dissection. Control: age-matched organ donors undergoing heart transplant surgery without AD, aneurysm, coarctation, or previous aortic repair. TG, triglyceride; LDL, low-density lipoprotein cholesterol; HDL, high-density lipoprotein cholesterol.

**Table S6. gRNA sequence**

| gRNAs | Sequence (5’-3’) |
| --- | --- |
| gRNA1 | CTGCTGATTGGAGAAACCAA AGG |
| gRNA2 | CACTGGACGGAAAGTACCTT TGG |
| gRNA3 | TGCCTTCGAAAGACCCCAAC AGG |
| gRNA4 | GAAGTCAGTGGCCGACCTGT TGG |

Primer information

| Primer Type | Sequence (5’-3’) |
| --- | --- |
| Forward | ACCAGGCAAAACAGAACCCA |
| Reverse | AAGCCCTCTCTCTGTTTGCC |

Primer information for identifying F0 generation mice

**Table S7. gRNA sequence**

| gRNAs | Sequence (5’-3’) |
| --- | --- |
| gRNA1 | GGCTAGATCTAGATGAGGAA AGG |
| gRNA2 | AGTTAGGCTAGATCTAGATG AGG |
| gRNA3 | CCAGAATGTTCCTGGTATGG TGG |
| gRNA4 | CAGAATGTTCCTGGTATGGT GGG |

Primer information for identifying CCDC80^fl/fl^ mice

| Primer Type | Sequence (5’-3’) |
| --- | --- |
| Forward | CTCCTTGGGCTCAGTGTGAC |
| Reverse | TTCCTATTTGTGGGCACTCAG |

Primer information for identifying *SM22α-Cre* mice

| Primer Type | Sequence (5’-3’) |
| --- | --- |
| Forward | CTCTCCTTGGGCTCAGTGTG |
| Reverse | CAAGAGGACCCAGGAACCAG |

**Table S8. Information regarding RT-PCR sequences**

| Gene | Primer | Sequence (5’-3’) |
| --- | --- | --- |
| CCDC80 (mouse) | Forward | AGCGAGCTGAGGAAGGAGTA |
|  | Reverse | ACGGACTTCATTGCTATTGGC |
| CDC80 (human) | Forward | GAAGACGTACCAGCCCATTT |
|  | Reverse | GGGAAGGATACCAGGATTTGAC |
| GAPDH (mouse) | Forward | CCCATGTTTGTGATGGGTGTG |
|  | Reverse | TGGCATGGACTGTGGTCATGA |
| GAPDH (human) | Forward | GTCATCCCTGAGCTGAACGG |
|  | Reverse | TGGGTGTCGCTGTTGAAGTC |
| α-SMA (mouse) | Forward | CACCATTGGAAACGAACGCT |
|  | Reverse | GTACTTGCGTTCTGGAGGGG |
| SM22α (mouse) | Forward | ACGATGGAAACTACCGTGGAG |
|  | Reverse | TTGAAGGCCAATGACGTGCT |
| MYH11(mouse) | Forward | TCTATGCCATTGCCGACACA |
|  | Reverse | GATGCCACCACAGCCAAGTA |
| CNN1(mouse) | Forward | GCGTCACCTCTATGATCCCAA |
|  | Reverse | CCCAGACCTGGCTCAAAGAT |
| OPN (mouse) | Forward | AATCTCCTTGCGCCACAGAA |
|  | Reverse | TGCTTGGAAGAGTTTCTTGCT |
| PCNA (mouse) | Forward | TTTGAGGCACGCCTGATCC |
|  | Reverse | GGAGACGTGAGACGAGTCCAT |
| P21 (mouse) | Forward | CGAGAACGGTGGAACTTTGAC |
|  | Reverse | CCAGGGCTCAGGTAGACCTT |
| Cyclin A2 (mouse) | Forward | CTTGGCTGCACCAACAGTAA |
|  | Reverse | CAAACTCAGTTCTCCCAAAAACA |
| TNFα (mouse) | Forward | TGTACCTTGTCTACTCCCAGGT |
|  | Reverse | ATAGCAAATCGGCTGACGGT |
| CCL2 (mouse) | Forward | CAGGTCCCTGTCATGCTTCT |
|  | Reverse | GTGGGGCGTTAACTGCATCT |
| MMP2 (mouse) | Forward | CCTGACCTGGACCCTGAAAC |
|  | Reverse | TCCCAGCGTCCAAAGTTGAT |
| MMP9 (mouse) | Forward | CAGCCACCACCACAACTGAA |
|  | Reverse | ATTGCAAGGATTGTCTGCCG |
| MMP13 (mouse) | Forward | GATGACCTGTCTGAGGAAGACC |
|  | Reverse | CTCTGGAACCTGAGACATCACC |
| MMP14 | Forward | GGATGGACACAGAGAACTTCGTG |
|  | Reverse | CGAGAGGTAGTTCTGGGTTGAG |


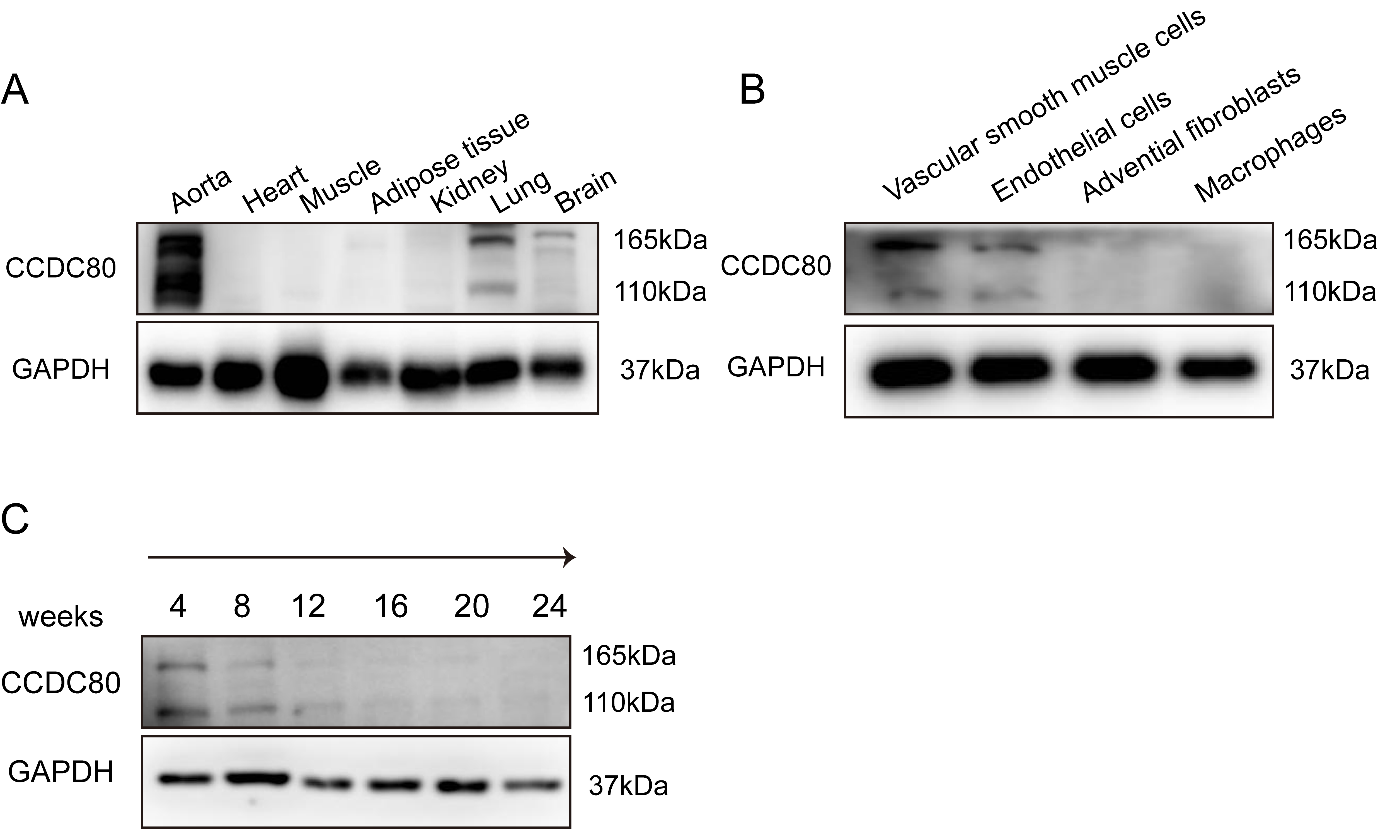


**Figure S1. Expression of CCDC80 in different tissues and vascular cells**

(A) The tissues (aorta, heart, muscle, adipose tissue, lung, and brain) of male C57BL/6J mice aged 10 weeks were harvested. CCDC80 protein expression was measured by western blotting. (B) CCDC80 expression in four vascular cell types: mouse primary vascular smooth muscle cells (VSMCs), mouse primary vascular endothelial cells, mouse vascular adventitial fibroblasts, and mouse macrophages. (C) Aortic tissues of male C57BL/6 mice aged 4, 8, 12, 16, 20, and 24 weeks were harvested. CCDC80 protein expression was measured by western blotting.


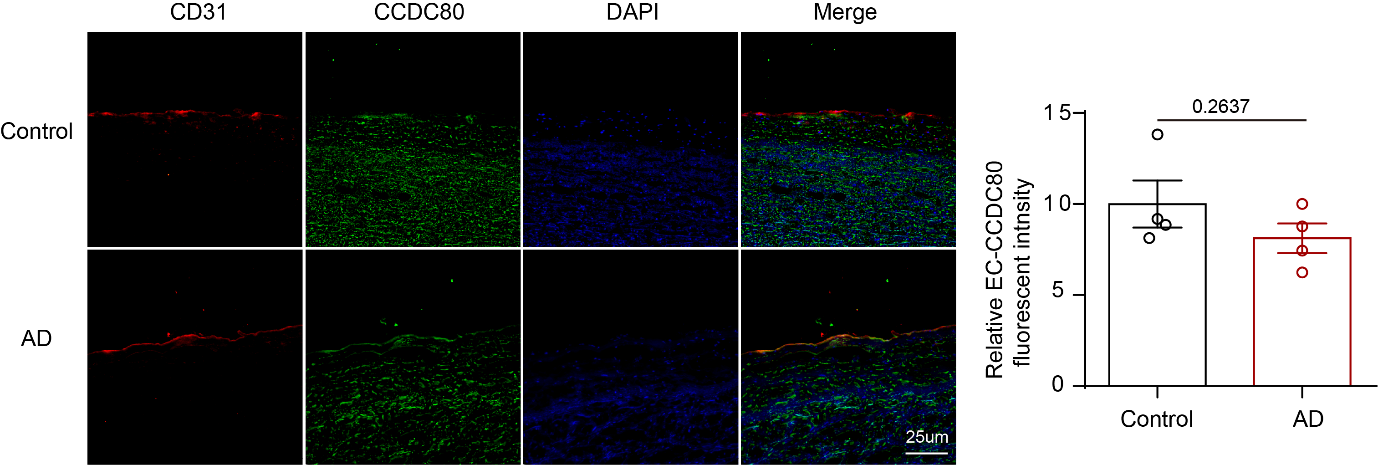


**Figure S2. Expression of CCDC80 in vascular endothelial cells during AD**

CCDC80 expression was not reduced in vascular endothelial cells during AD. Representative immunofluorescence staining CCDC80 (green) and CD31 (red) in human normal and AD aortas. Quantification of the CCDC80-positive area in endothelial cells in the panel on the right, n = 4 per group. Data are presented as mean ± SEM. Statistical analysis was performed using Student’s *t*-test.


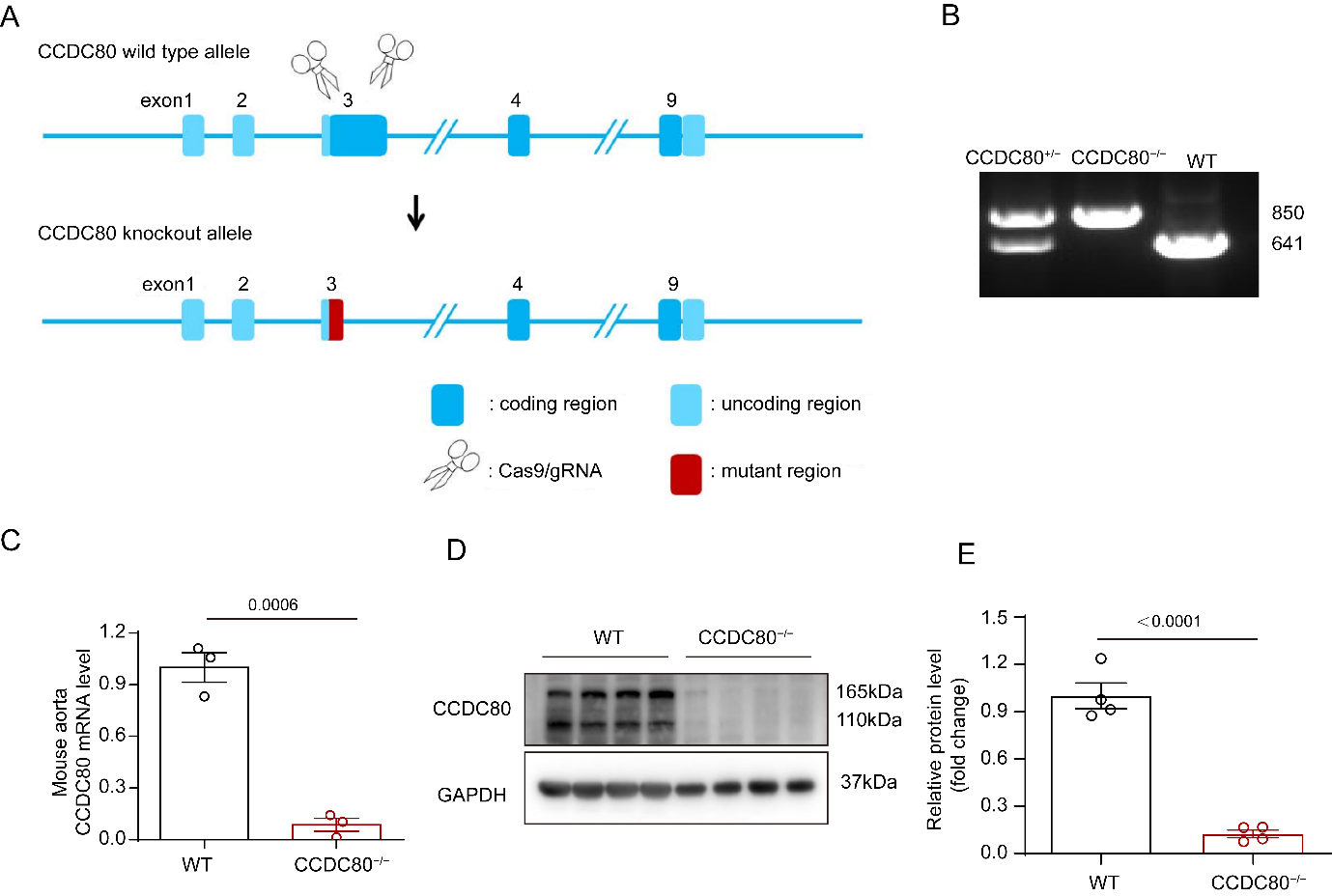


**Figure S3. Identification of CCDC80-deficient mice**

(A) Schematic of the strategy used for CCDC80 gene editing. (B) PCR analysis for the validation of gene editing. The 850-bp band represents CCDC80^−/−^. The 641-bp band represents the wild type. (C) The expression of CCDC80 mRNA levels in aortic tissues was determined using qPCR in the indicated groups, n = 3 per group. (D) The expression of CCDC80 protein in aortic tissues was determined by western blotting in the indicated groups, n = 4 per group. Data are presented as mean ± SEM. Statistical analysis was performed using Student’s t-test for C and E.


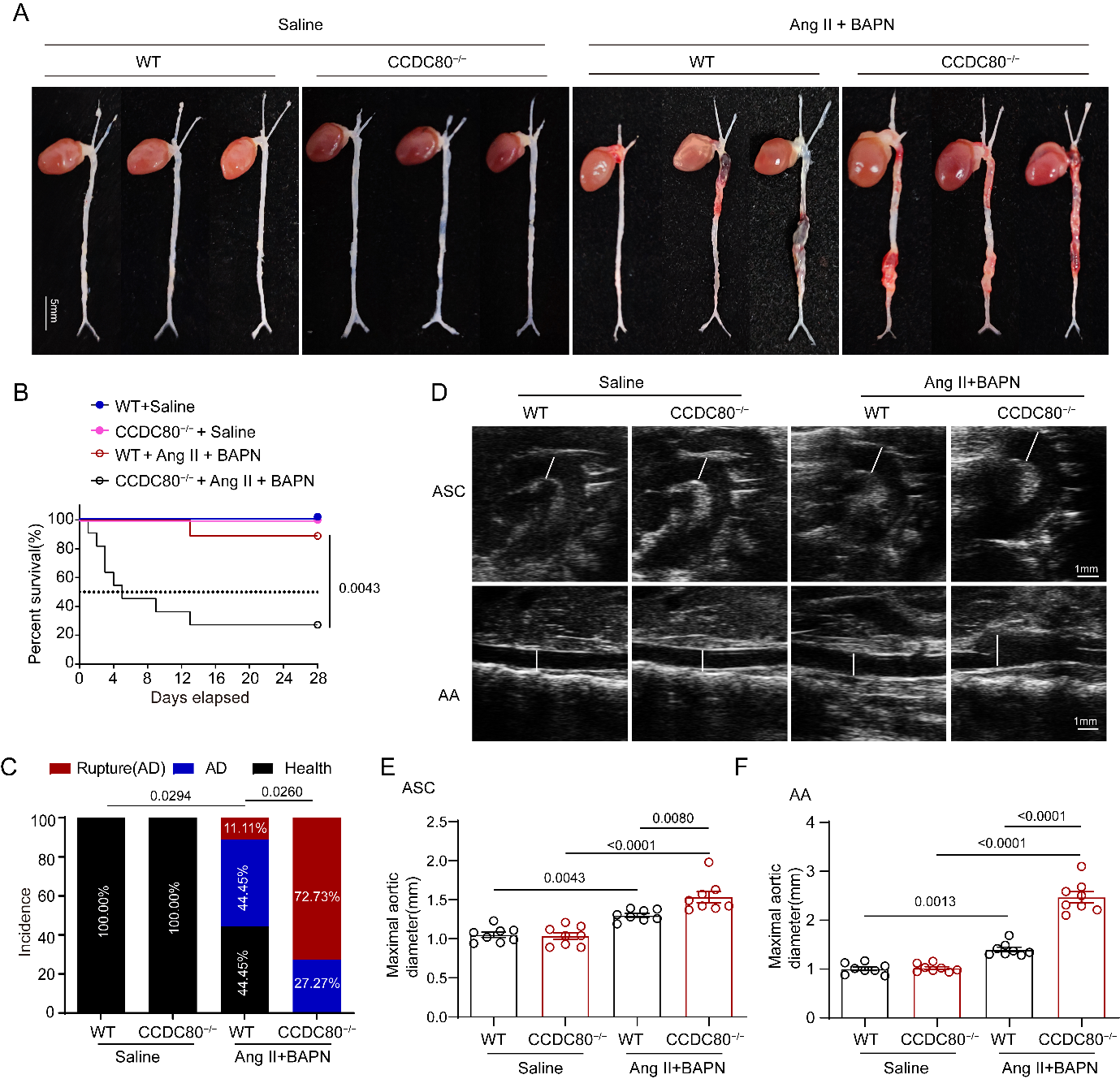


**Figure S4. Aggravation of Ang II + BAPN-induced AD by CCDC80 deletion**

(A–F) WT and CCDC80^−/−^ mice were treated with saline or angiotensin II (Ang II) + β-aminopropionitrile monofumarate (BAPN) for 28 d. (A) Representative images of aortas from WT and CCDC80^−/−^ mice treated with saline or Ang II + BAPN for 28 d. (B) Kaplan–Meier survival curves of male WT and CCDC80^−/−^ mice during 14 days of saline or Ang II + BAPN administration, n = 9-11 per group. (C) Quantification of incidence (AD, rupture, and Health) in whole aortas from WT and CCDC80^−/−^ mice. (D) Representative ultrasound images of the ascending aorta (ASC) and abdominal aorta (AA); scale bar = 1 mm. (E and F) Measurements of maximum ASC and AA, WT mice and CCDC80^−/−^ mice, n = 8 per group. Data are presented as mean ± SEM. Statistical analysis was performed by using the Kaplan–Meier method and compared using log-rank tests for B, A Fisher’s exact test for C, and 2-way ANOVA with Tukey’s post hoc test for E and F.


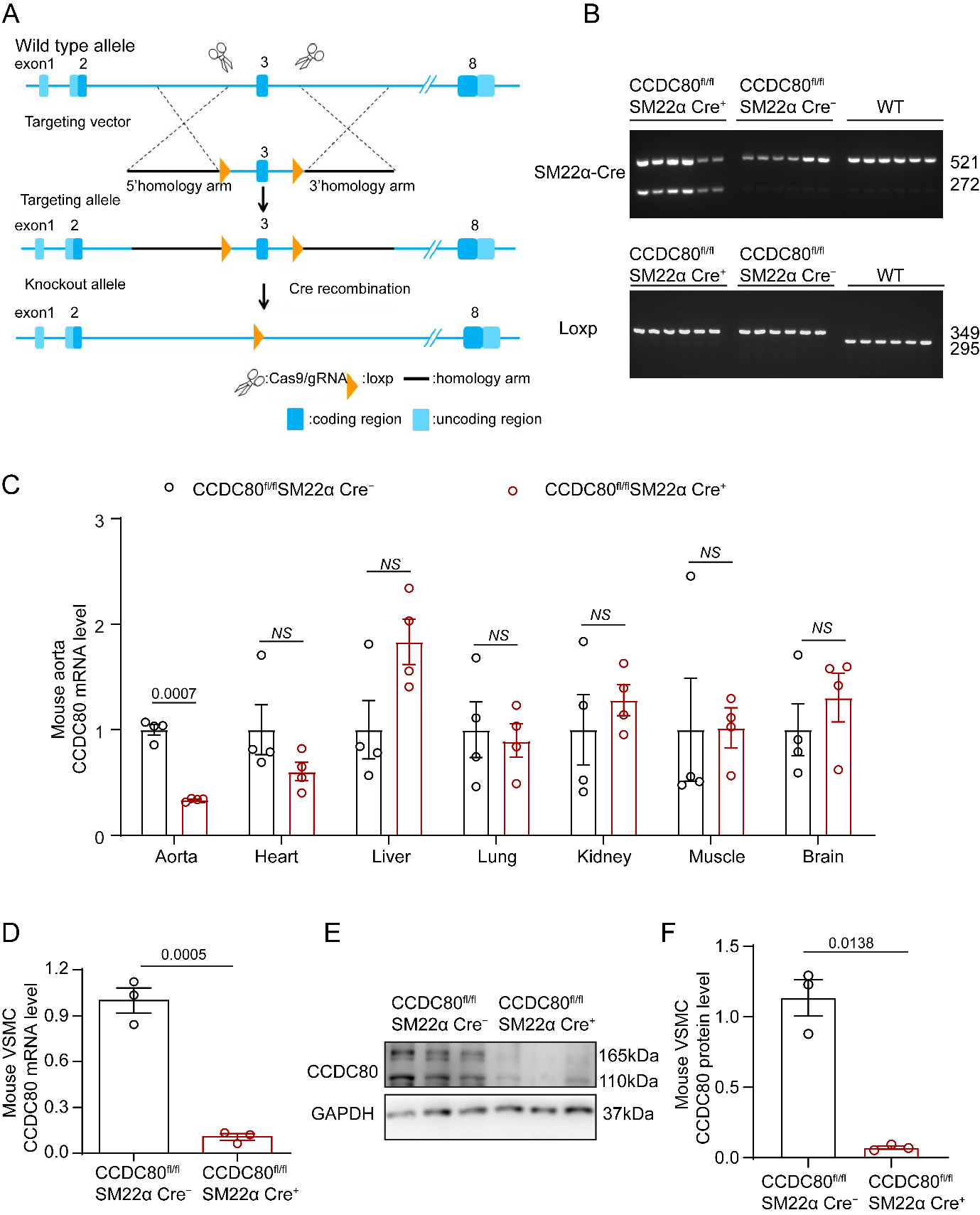


**Figure S5. Identification of VSMC-specific CCDC80-deficient mice**

(A) Schematic of the CCDC80 gene editing strategy. To generate VSMC-specific CCDC80-deficient mice (CCDC80^fl/fl^ SM22α Cre^+^), CCDC80^fl/fl^ mice were crossed with mice carrying the SM22α Cre transgene. The littermate CCDC80^fl/fl^ SM22α Cre^−^ mice were used as controls. (B) PCR analysis for the validation of gene editing. The 272-bp band represents SM22α Cre. The 349-bp band represents the mutation resulting from the insertion of LoxP in introns 2 and 4. The 295-bp band represents the wild type. (C) The expressions of CCDC80 mRNA in different tissues (aorta, heart, liver, lung, kidney, muscle, and brain) were determined by qPCR in the indicated groups, n = 4 per group. (D–F) The expression of CCDC80 mRNA and proteins in VSMCs were determined by qPCR (D) and western blotting (E, F) in the indicated groups, n = 3 per group. Data are presented as mean ± SEM. Statistical analysis was performed using Student’s t-test [C(lung), C(kidney), C(liver), and D), Student’s t-test with Welch’s correction (C(aorta) and F), the Mann-Whitney U test [C(heart), C(muscle) and C(brain)]. *NS*, statistically nonsignificant.


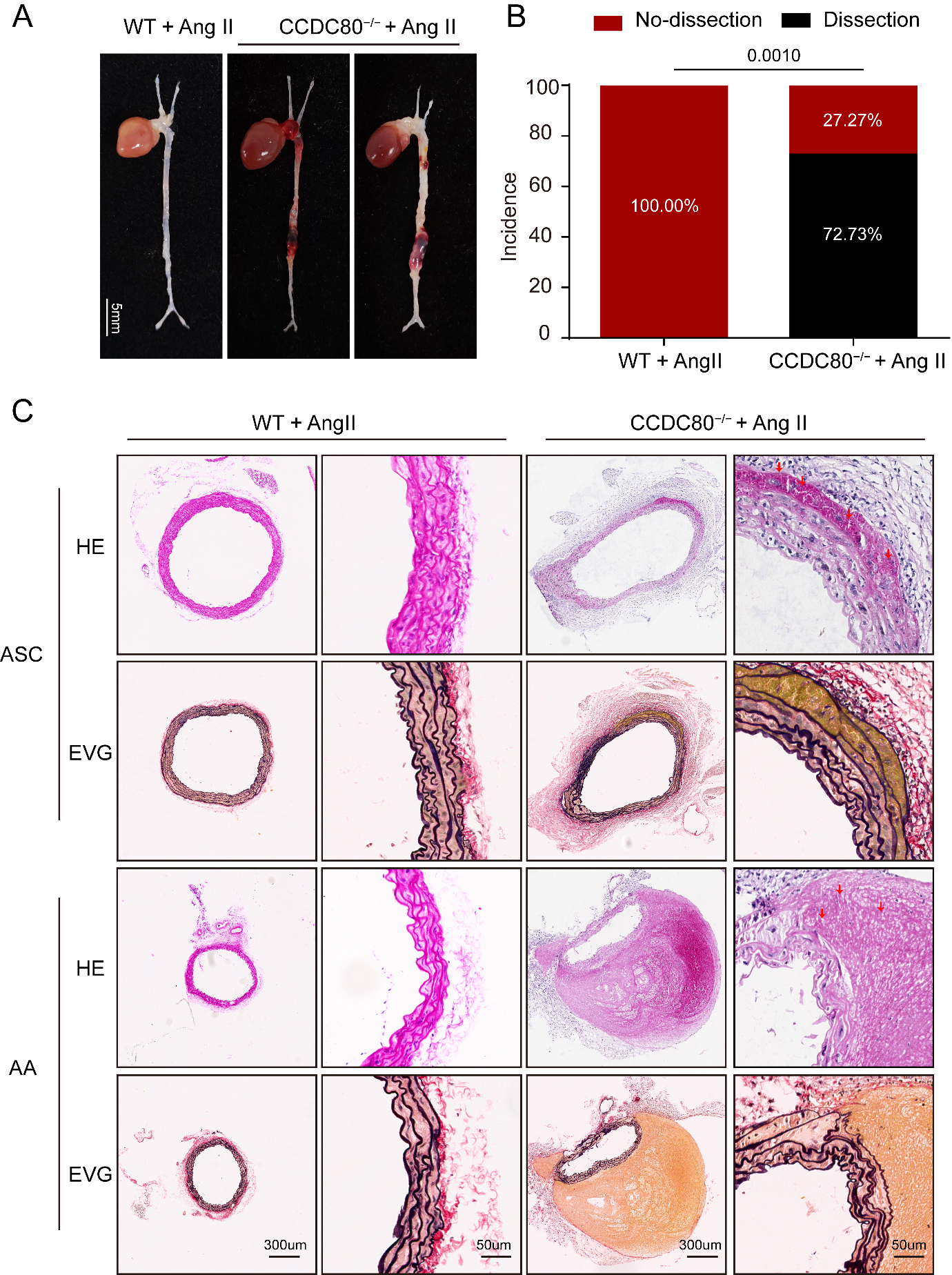


**Figure S6. Induction of AD by CCDC80 deletion on day 3 after angiotensin II (Ang II) administration**

(**A**) Representative pictures of whole aortas of WT (n = 11) and CCDC80^−/−^ (n = 11) mice on day 3 after Ang II administration. (**B**) Quantification of incidence (No dissection and dissection) in whole aortas from WT and CCDC80^−/−^ mice with 3 days of Ang II administration; statistical analysis was performed using a chi-square test. (**C**) Representative pictures of staining for H&E, EVG, and Masson’s staining techniques in the ascending aortas and abdominal aortas of WT and CCDC80^−/−^ mice at day 4 after Ang II administration. Red arrows indicate red blood cells. Statistical analysis was performed using A Fisher’s exact test for B.


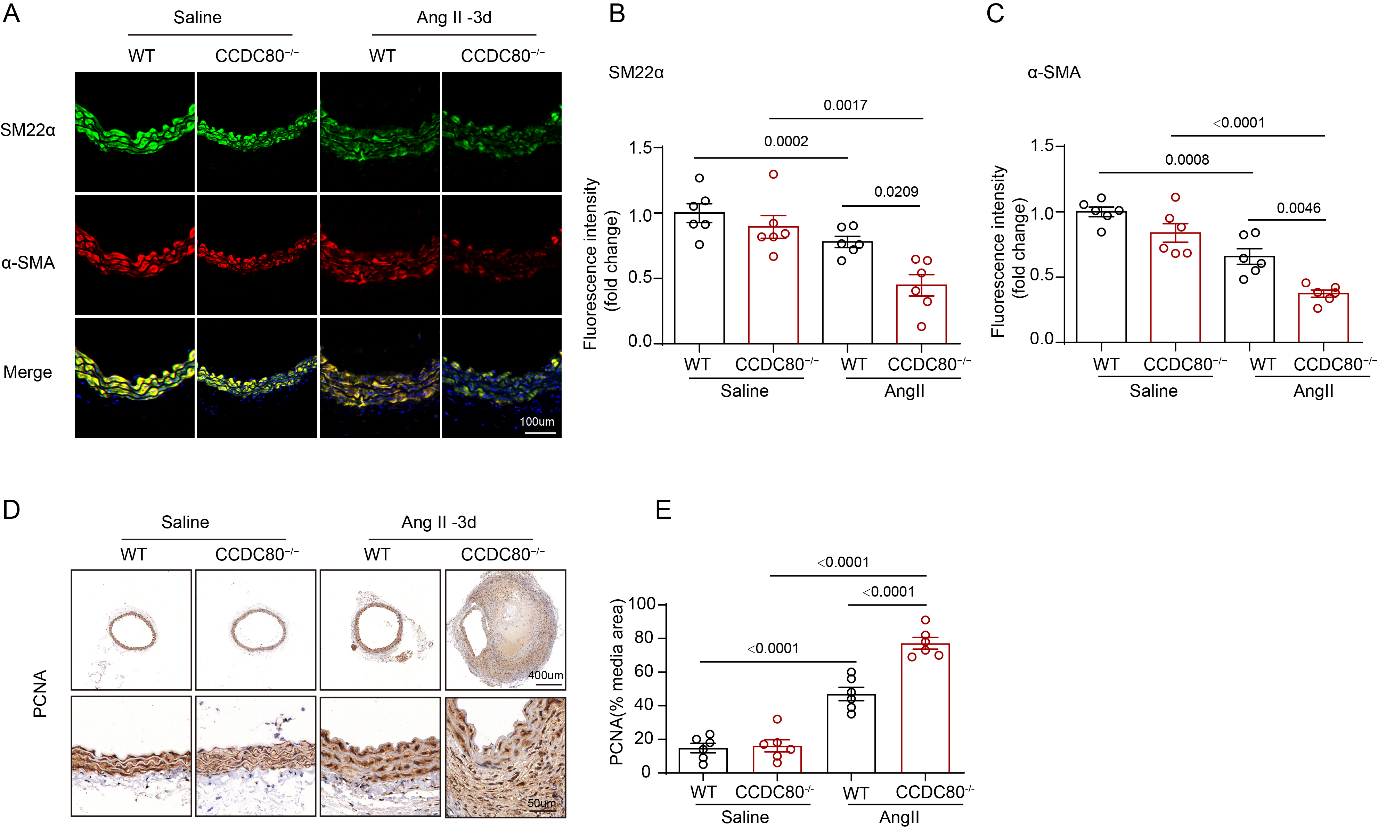


**Figure S7. Ang II-induced synthetic marker expression in VSMCs caused by CCDC80 deficiency**

(A–C) Immunofluorescence staining of SM22α (green) and α-SMA (red) of the abdominal aortas (AAs) from WT and CCDC80^−/−^ mice after 3 days of Ang II administration. Nuclei were stained with DAPI (blue); scale bar = 100 μm. Quantification of α-SMA and SM22α-positive areas in aortas, n = 6 per group. (D) Representative images of PCNA expression by immunohistochemical staining in mouse AAs. (E) Quantification of PCNA-positive area in aortic media, n = 6 per group. Data are presented as mean ± SEM. Statistical analysis was performed using 2-way ANOVA with Tukey’s post hoc test for B, C, and E.


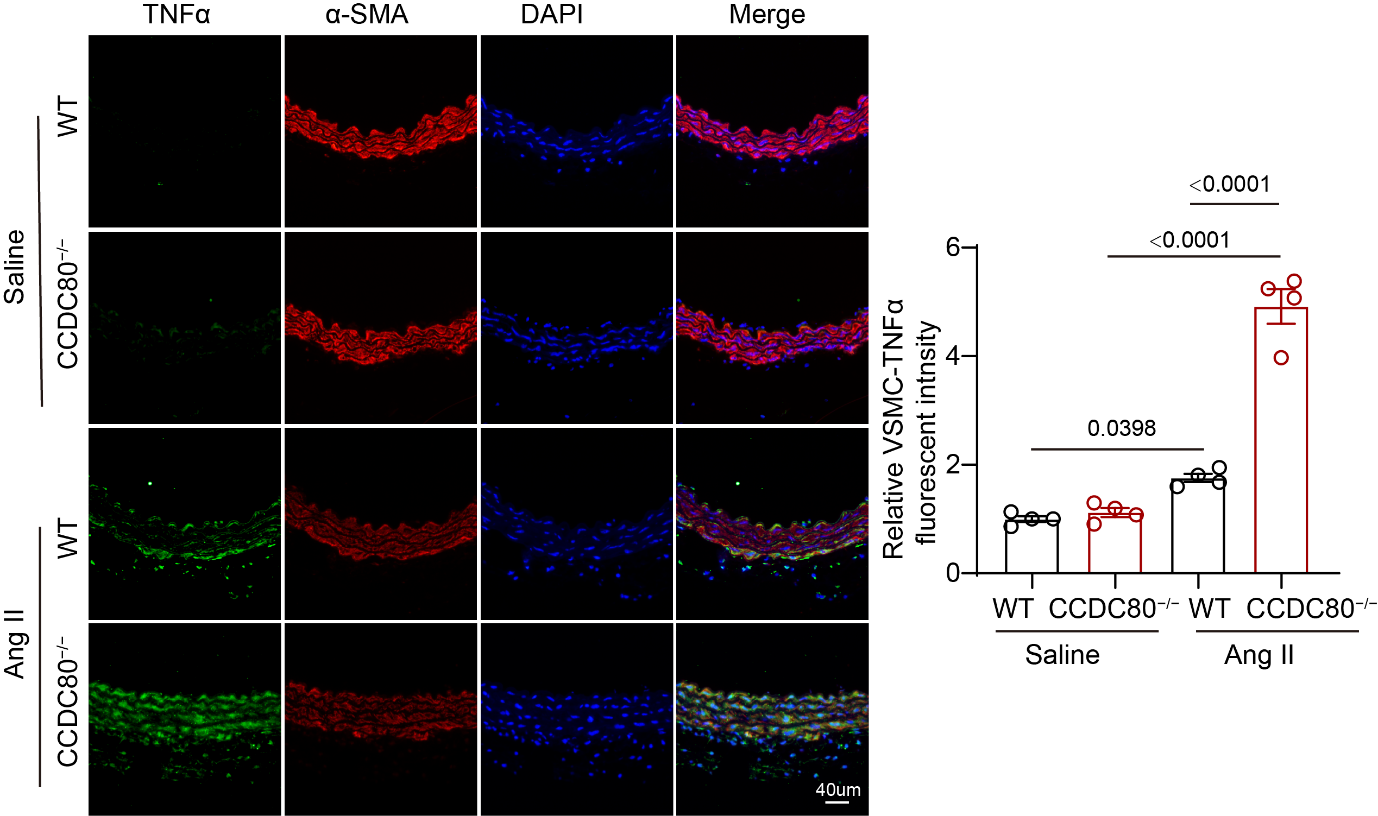


**Figure S8. Enhanced proinflammatory response by synthetic VSMCs mediated by CCDC80 deletion**

Representative immunofluorescence staining of TNFα (green) and α-SMA (red) in ascending aortas from WT and CCDC80^−/−^ mice after 3 days of angiotensin II (Ang II) administration; scale bar = 40 µm. Quantification of TNFα-positive area in aortic media in the right panel, n = 4 per group. Data are presented as mean ± SEM. Statistical analysis was performed using 2-way ANOVA with Tukey’s post hoc test.


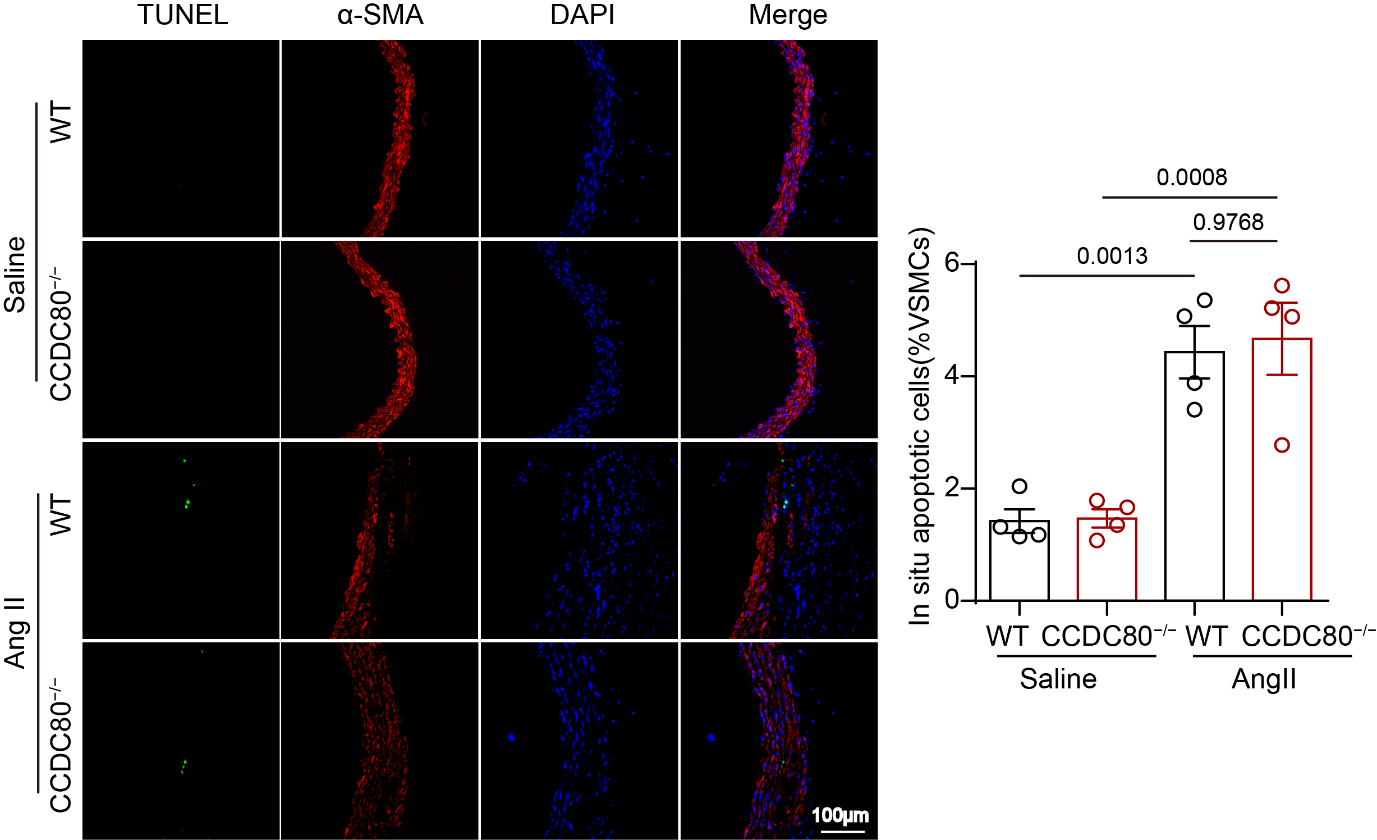


**Figure S9. Effect of Ang II on VSMC apoptosis** **in CCDC80^−/−^ mice**

Ang II exerts no effect on VSMC apoptosis in CCDC80^−/−^ mice. Representative immunofluorescence staining of TUNEL (green) in the aortas from WT and CCDC80^−/−^ mice after 3 days of Ang II administration; scale bar = 100 µm. Quantification of apoptotic cells in aortic media in the right panel, n = 4 per group. Data are presented as mean ± SEM. Statistical analysis was performed using 2-way ANOVA with Tukey’s post hoc test.


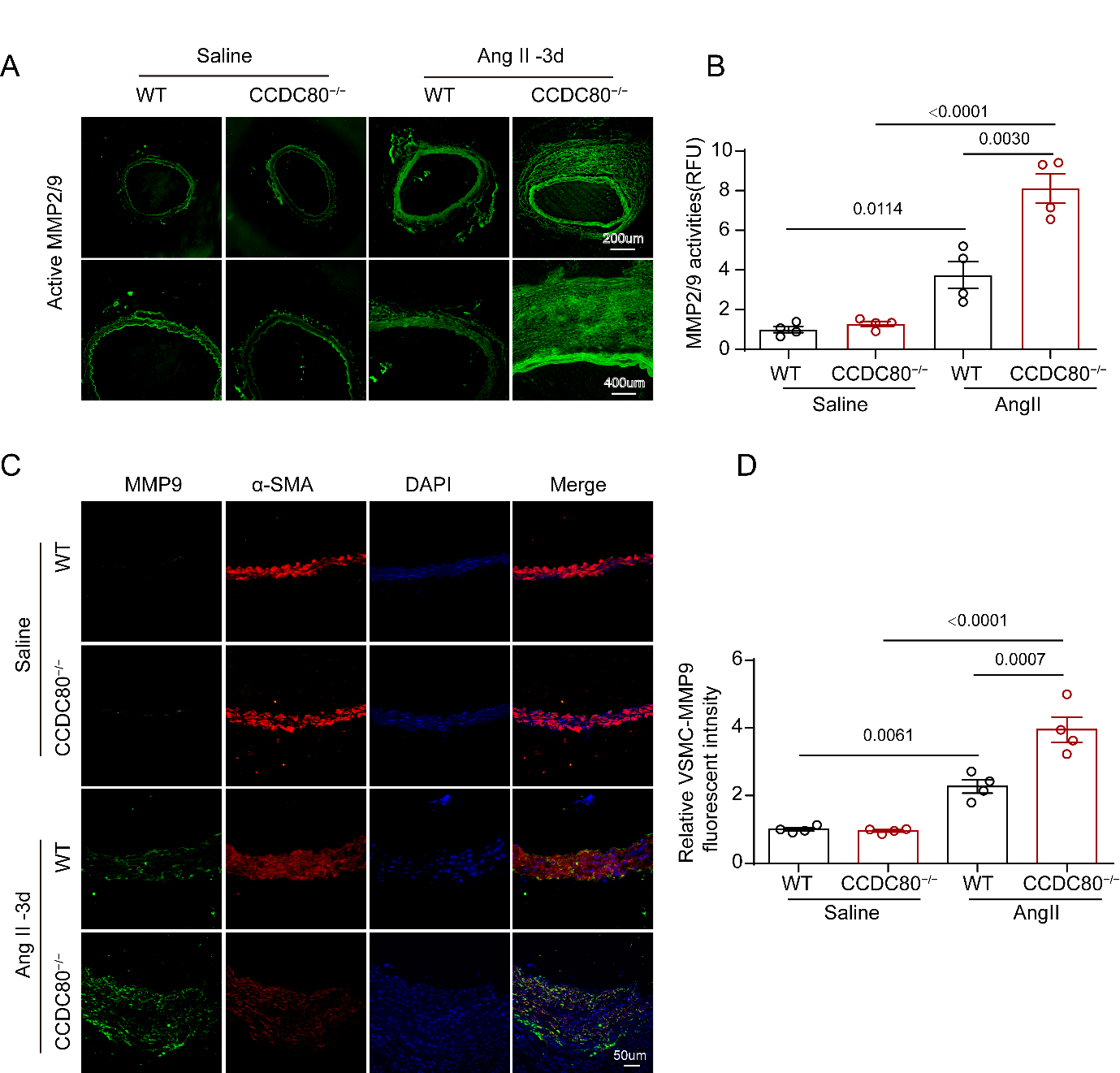


**Figure S10. CCDC80 deficiency enhances MMP production**

(A, B) In situ fluorescence zymography for MMP2/9 activity and quantification of the MMP2/9-positive area in the aortas, n = 4 per group. (C) Immunofluorescence staining of MMP9 (green) and α-SMA (red) in the ascending aorta. Nuclei were stained with DAPI (blue); scale bar = 50 μm. Quantification of MMP9-positive area in aortic media in the right panel, n = 4 per group. Data are presented as mean ± SEM. Statistical analysis was performed using 2-way ANOVA with Tukey’s post hoc test.


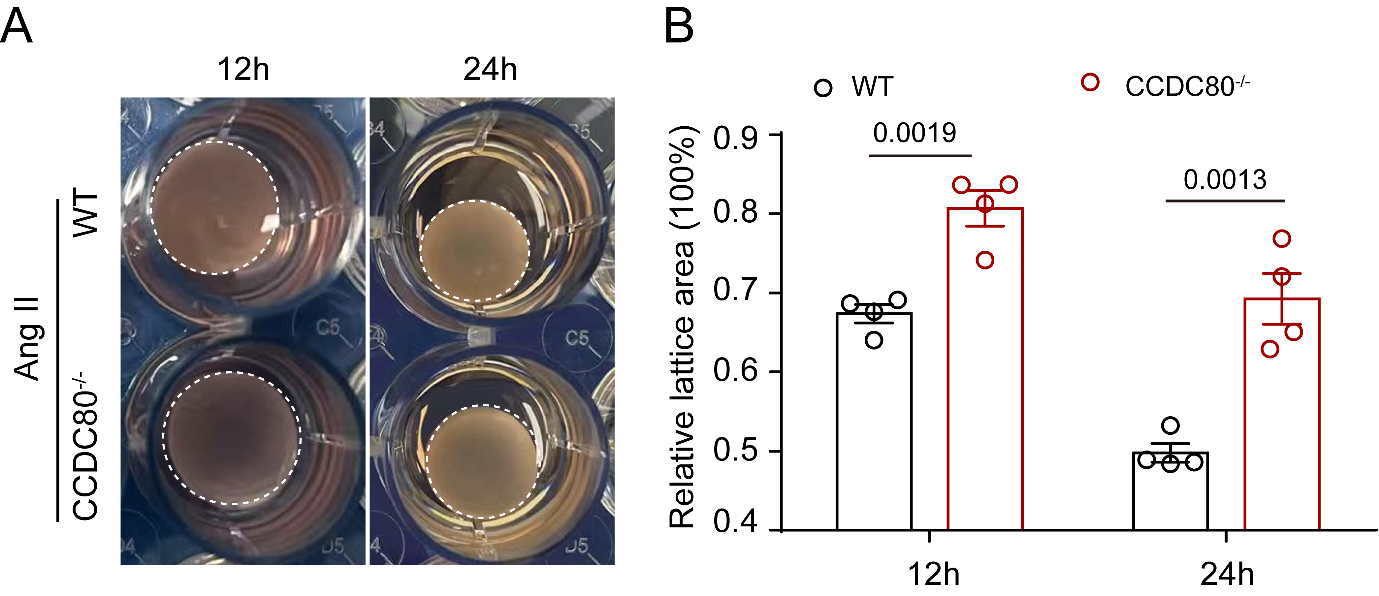


Figure S11. CCDC80 deficiency inhibits Ang II-induced VSMC contraction in primary VSMCs in vitro

(A, B) Collagen-based contraction assay in mouse VSMCs isolated from WT and CCDC80^−/−^ mice. VSMCs were harvested and mixed with ice-cold collagen gel solution together with saline or Ang II (1 μM) (n = 4 independent experiments). Data are presented as mean ± SEM. Statistical analysis was performed using Student’s t-test.


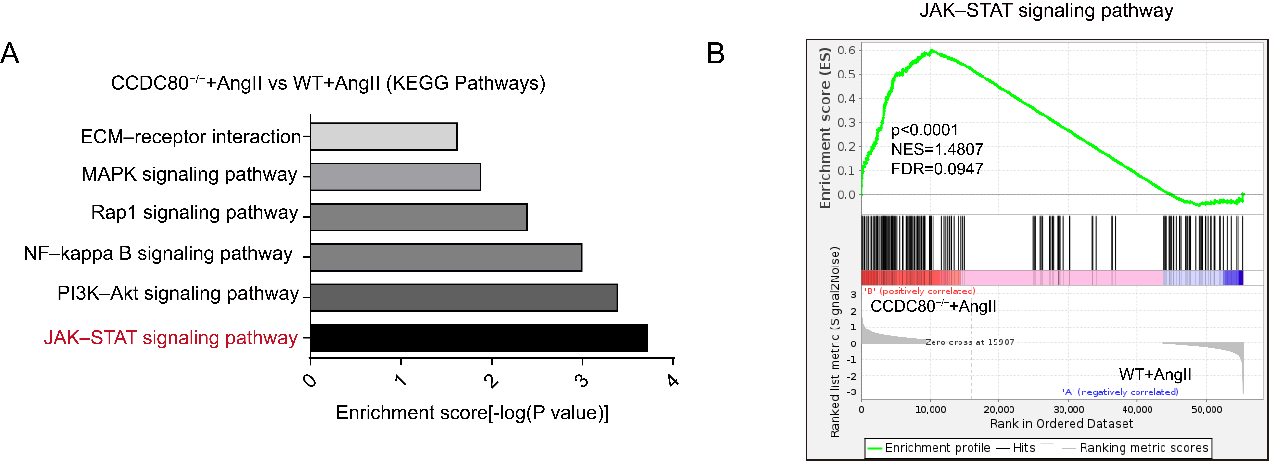


**Figure S12. CCDC80 knockout-mediated activation of the JAK/STAT pathway**

(A) KEGG pathway analysis of DEGs of aortic tissues from WT and CCDC80^−/−^ mice after 3 days of Ang II administration. (B) Gene-set enrichment analysis showing a positive correlation between CCDC80 deletion and JAK/STAT signaling pathway.


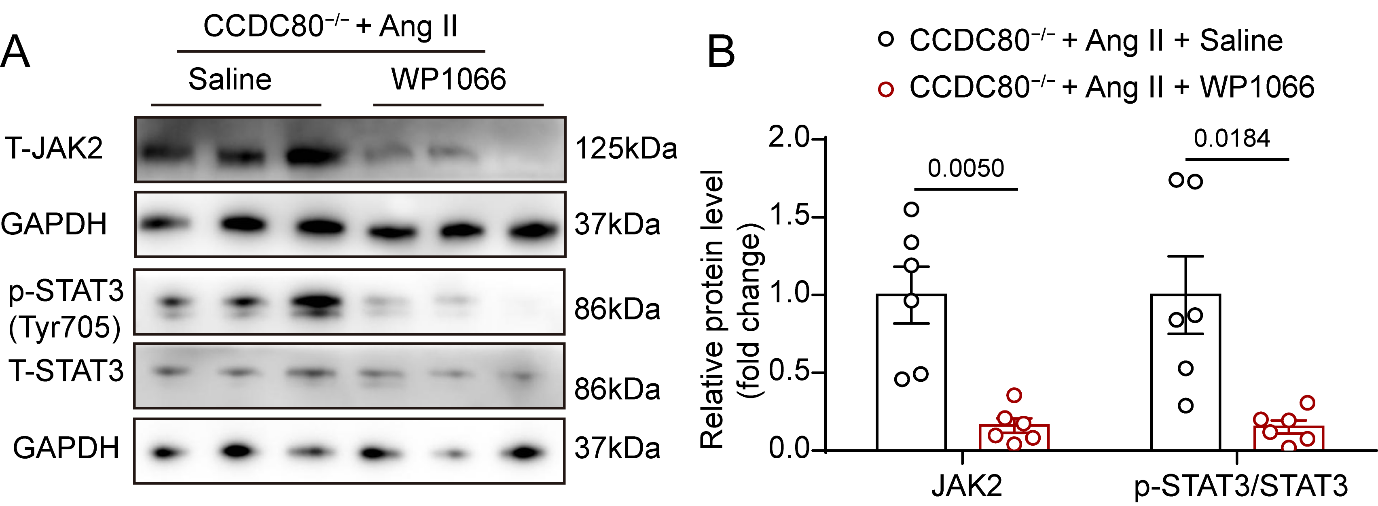


Figure S13. WP1066 inhibits the JAK2/STAT3 pathway

(A, B) CCDC80−/− mice were treated with WP1066 or saline after Ang II administration for 14 days. Western blotting and quantification of JAK2, STAT3, and p-STAT3 (Tyr705) in aortic tissues (n = 6 per group). Data are presented as mean ± SEM. Statistical analysis was performed using Student’s t-test with Welch’s correction.


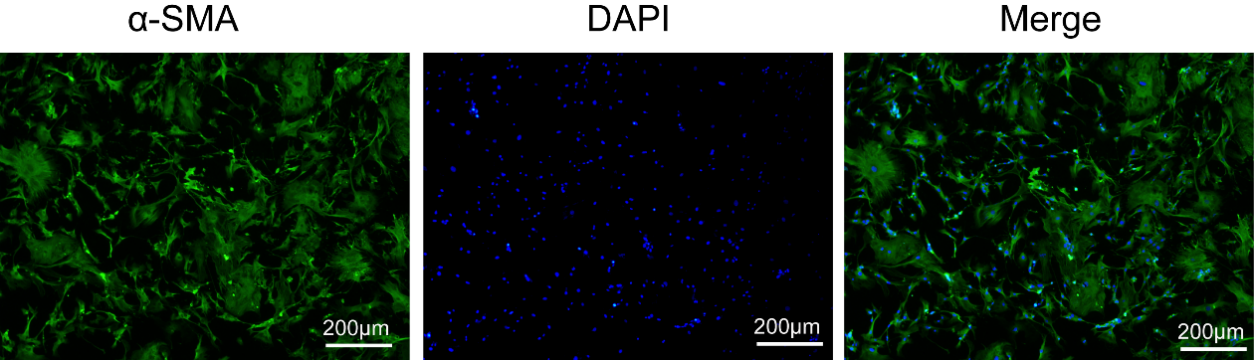


Figure S14. Identification of primary vascular smooth muscle cells (VSMCs)

VSMCs were isolated from mice and then positively identified by staining α‐smooth muscle actin (α‐SMA).
